# Expression pattern of the 10 mitogen-activated protein kinase kinases (MAPKK) encoded on *Arabidopsis thaliana* genome

**DOI:** 10.1080/15592324.2026.2697589

**Published:** 2026-07-06

**Authors:** Salvador Barrera-Ortiz, José Manuel González-Coronel, Yessica Casales-Tlatilpa, Jesús Salvador López-Bucio, Julián David Camargo-Pacanchique, Ángel Arturo Guevara-García

**Affiliations:** a Instituto de Biotecnología, Departamento de Biología Molecular de Plantas. Universidad Nacional Autónoma de México, Cuernavaca, Morelos, México; b Laboratorio de Biología Molecular, Centro de Desarrollo de Productos Bióticos, Instituto Politécnico Nacional, Yautepec, Morelos, México; c Investigador por México SECIHTI-UMSNH, Instituto de Investigaciones Químico Biológicas, Universidad Michoacana de San Nicolás de Hidalgo, Morelia, Michoacán, México; d Pontificia Universidad Javeriana, Bogotá, Colombia

**Keywords:** Post-embryonic development, plant signaling, MAP kinase modules, *cis*-regulatory elements

## Abstract

Phosphorylation, carried out by kinases, is a pivotal mechanism for all cell signal transduction pathways. In eukaryotic cells, Mitogen-Activated Protein Kinases (MAPK) form modules with three kinases (MAPKKK, MAPKK, and MAPK) that are sequentially activated, participating in multiple signaling mechanisms. The second kinase forming a typical module is called MAP kinase kinase (MAPKK or MKK), which receives the signal from MAPKKK and transmits it to MAPK. The *Arabidopsis thaliana* genome has 10 MKKs and they are classified into four different groups (A, B, C, and D). To date, several aspects of the MKK functioning are known, but there is little information about *MKK* expression in plant tissues. Here, each *MKK* upstream region was fused to the *UidA* reporter, and these *pMKK1-10:UidA* constructions were used to reveal the *MKK* expression patterns in the tissues of the seedlings during their early development. This experimental expression analysis was complemented with a detailed bioinformatic analysis focused on identifying the *cis*-regulatory elements present in these upstream promoter regions.

## Introduction

To contend with the changing conditions of nature, plants reprogram the expression of their genes.^1^ Mitogen‐activated protein kinase (MAPK) cascades are key signaling modules downstream of receptors/sensors. MAPK modules coordinate cellular responses to achieve normal growth/development of organisms and their adaptation to the ever‐changing environment.^2^ Via a sequential phosphorelay mechanism, these cascades have three types of kinases that are activated in a hierarchical order (MAPKKK, MAPKK, and MAPK), linking upstream receptors to downstream targets.^3^

Plant MAPKKs (or MKKs) are dual‐specificity kinases that phosphorylate MAPKs on both the Thr (T) and Tyr (Y) residues in the TXY motif, and they are phosphorylated in the consensus sequence S/T-XXXXX-S/T of the activation loop by MAPKKKs.^2^ Viridiplantae MKKs are classified into five groups (A-E), and the *Arabidopsis thaliana* genome contains 10 genes encoding MKKs which belong to any of the A, B, C or D groups.^4^

Group A includes MKK1, MKK2, and MKK6, which have been implicated with the response to abiotic and biotic stresses.^2^ ^,^ ^5^ MKK3 is the only member of group B and participates in cascades that are elicited by pathogens in a hormone dependent manner.^6^ ^,^ ^7^ Group C includes MKK4 and MKK5, which play redundant functions in plant immunity and growth/development.^2^ ^,^ ^8^ The remaining MKK7, MKK8, MKK9, and MKK10 form group D, which is the least studied. However, previous studies have shown that MKK7 is involved in the response to pathogens by a salicylic acid-dependent mechanism.^9^ Additionally, it is known that MKK9 participates in ethylene signaling, by regulating EIN3 stability.^10^ Finally, MKK10 regulates red-light controlled seedling photomorphogenesis.^11^

Interestingly, the number of *MAPKKs* is usually much smaller than that of *MAPKs* and *MAPKKKs* in different species. 20 MAPKs and 80 MAPKKKs are encoded in the *A. thaliana* genome.^4^ The above suggests that MPKKs are nodes where multiple signals converge and control the functions of several MAPKs. Then, MAPK modules may share kinase components, but their signaling specificity is maintained by spatiotemporal constraints and dynamic protein‒protein interactions.^5^

In this work, we generated *UidA* transgenic lines of the 10 MKKs of *A. thaliana*, and evaluated their expression patterns in different parts of the seedlings during post-embryonic growth. The *UidA* expression of all the transgenic lines was modified in an age-dependent manner, and except for *pMKK5:UidA* and *pMKK10:UidA*, this expression was widely distributed in plant tissues. *pMKK10:UidA* was not found in the root, while *pMKK3:UidA* and *pMKK4:UidA* were even found in the root meristem. Additionally, our study was complemented with a comparative bioinformatic analysis of *cis* regulatory elements present in the *MKK* regulatory regions. Since the *cis* regulatory elements of the promoters bind to *trans* regulatory factors by determining the levels and patterns of gene expression. These findings, with subsequent research using the transgenic lines of this study, will allow us to elucidate how the transcriptional regulation of MKKs is carried out in plants.

## Materials and methods

### Plant individuals, materials, and experimental conditions

*A. thaliana* wild-type (Wt) seeds of ecotype Columbia 0 (Col-0) were used for all the experiments. The Col-0 seeds were cleaned from the surface with a first treatment of industrial ethanol [96°] in agitation [1400 rpm] for 4 min, and a second treatment with 10% [v/v] of commercial chlorine in agitation [1400 rpm] for 4 min. After 5 washes with sterile deionized water, they were incubated with another aliquot of that water at 4 °C during 48 h. Wt seeds were sown on solidified media inside Petri dishes, which were incubated at 22 °C into a plant growth chamber (Percival CU41L4) with a photoperiod of 16 h of light and 8 h of darkness. The medium was made up of 0.09% [w/v] of Murashige and Skoog (MS) basal salt mixture, 0.6% [w/v] of sucrose, and 1% [w/v] of plant tissue-culture agar (Phytagar), being adjusted to pH 7 with 10% [w/v] of KOH before the Phytagar was added. The MS medium used in this study was made at 20% of the tobacco formulation and was designated 0.2X MS medium. MS basal salt mixture, sucrose, and Phytagar were purchased in Phytotechnology Laboratories®, and KOH was purchased in J. T. Baker®.

### Construction of *pMKK1-10:UidA* transgenic lines

**Figure 1.**
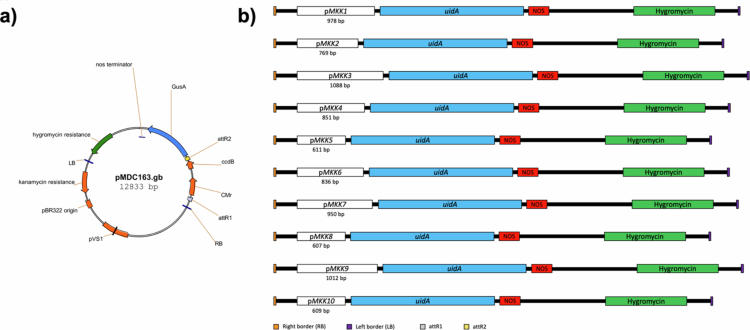
Schematic representation of the binary vector pMDC163 and transcriptional fusions from *Arabidopsis thaliana MKK1-10* promoters with the sequence encoding beta-glucuronidase (*UidA*). a) The binary vector pMDC163 were used to make. b) transcriptional fusions from the promoter regions of ten mitogen-activated protein kinase kinase (MPKK) encoded in the *Arabidopsis thaliana* genome, with open reading frame of *UidA*.

The transgenic lines were made in similar way to *pMPK6:UidA*, as previously reported by López-Bucio and coworkers.^12^ The 10 upstream intergenic regions of the 10 *MAP Kinase Kinase* (*MAPKK* or *MKK*) genes of *A. thaliana* were amplified by PCR from genomic DNA with the primers: 5′ CAC CTA ATT CAG AAA GCT TCG 3′ (forward) and 5′ GTC TGG TTT TTG GAG ATC 3′ (reverse) for *pMKK1*; 5′ CAC CCG CAA TTT GTG ATA GGA 3′ (forward) and 5′ CGT CTT CGT CTA CTT CGT 3′ (reverse) for *pMKK2*; 5′ TAG AGT TCG GAC GAT ACG 3′ (forward) and 5′ GAT ACC TAC TTT TTC TGT 3′ (reverse) for *pMKK3*; 5′ GGA GCT TGA GTG AAA AAC 3′ (forward) and 5′ GCT TTT GGA ATC AAA AGT 3′ (reverse) for *pMKK4*; 5′ CAC CGG CAA AGA AGG CAA ACT 3′ (forward) and 5′ GGC TTT TAG AAG AAG AGG 3′ (reverse) for *pMKK5*; 5′ CTT CTG GCA GCT CTT CTT 3′ (forward) and 5′ TTT TCT TTG GTT TCT TCC 3′ (reverse) for *pMKK6*; 5′ CTT TTG CAT GAT GGC CTA 3′ (forward) and 5′ TGG AAA TAG AAG AGA GAG 3′ (reverse) for *pMKK7*; 5′ CAA GTC CAG AGA CTG AAG 3′ (forward) and 5′ CTT GTT GTT GAA TGA TCG 3′ (reverse) for *pMKK8*; 5′ CCG TAA ACT TAA GGT TAT 3′ (forward) and 5′ TGG AAT AGA GAG AAG AAG 3′ (reverse) for *pMKK9*; 5′ CAC CTT TGT TCA GCT TCC TAG 3′ (forward) and 5′ TAG GTT ATT TGC TTG CAC 3′ (reverse) for *pMKK10*. The oligonucleotides were acquired from “Unidad de Síntesis y Secuenciación de DNA” of “Instituto de Biotecnología de la UNAM”. Ten gateway system entry clones were generated by cloning each of the amplified promoters into the pENTR/D-TOPO vector (Invitrogen™). The constructs were transferred to the destiny binary vector pMDC163 by LR recombination reaction (Figure 1). All the sequenced vectors containing *MKK1-10* promoters fused to *the UidA Escherichia coli* gene (*pMKK1-10:UidA*) were used to transform *Agrobacterium tumefaciens*, then the vectors were infiltrated into *A. thaliana* wild-type (Wt) plants (ecotype Col-0) via the floral-dip method.^13^ The expression patterns of three independent transgenic lines for each of the 10 constructions were evaluated, and the data confirmed that each group of three lines had similar expression patterns in both the root and shoot tissues.

### *UidA* expression by the β-glucuronidase assay

In this assay, the β-glucuronidase enzyme encoded in *UidA* reacted with the substrate 5-bromo-4-chloro-3-indolyl-β-D-glucuronide (X-Gluc), and formed a blue-colored product. *A. thaliana* seedlings carrying the *UidA* construct were incubated overnight at 37 °C in a microtiter plate with 0.1% [w/v] of X-Gluc in a buffer at pH 7, made up of 0.1 M NaH_2_PO_4_, 0.1 M Na_2_HPO_4_, 0.1% [v/v] Triton™ X-100, 10 mM EDTA, 2 mM K_4_Fe(CN)_6_, and 2 mM K_3_Fe(CN)_6_, to detect a blue coloration in the tissues. The X-Gluc buffer was removed, and the seedlings were incubated with 0.24 N of HCl in 20% [v/v] of methanol during 50 min at 62 °C. Subsequently, the acid solution was replaced by 7% [w/v] of NaOH in 60% [v/v] of ethanol during 20 min at room temperature. The basic solution was removed, and 40% [v/v] of ethanol was added during 20 min, which was substituted by 20% [v/v] of ethanol for 20 min, and the latter was replaced by 10% [v/v] of ethanol for 20 min. The last ethanol solution was discarded, and 50% [v/v] of glycerol was added. The seedlings were placed on glass slips with a parafilm compartment and 50% [v/v] of glycerol, covered with coverslips and sealed with commercial nail varnish, which was named “mounting”. Finally, the mountings were photographed with a Nomarski microscope (Leica DFC450C) at 10× magnification. The micrographs were taken from four regions of the seedling, and twelve individuals were analyzed at each time point. The analysis was conducted by focusing on the internal parts of the organs to show epidermis, cortex, endodermis, and stele tissues for roots and stems and mesophyll cells for cotyledons and leaves.

### Identification and analysis of *cis*-regulatory elements

#### Sequences under analysis

The upstream region sequences for the 10 members of the MKK family in *A. thaliana* (loci: *At4g26070*, *At4g29810*, *At5g40440*, *At1g51660*, *At3g21220*, *At5g56580*, *At1g18350*, *At2g06230*, *At3g73500*, *At1g32320,* and MKK1-10 respectively), were retrieved from the TAIR10 database (https://www.arabidopsis.org). The sequences range in length from 607 to 1088 bp upstream of each translation start codon (ATG), which is consistent with those previously used to generate the *pMKK1-10:UidA* reporter lines (Figure 1).

#### Identification of transcription factor binding sites

Transcription Factor Binding Sites (TFBS) on each upstream region were identified by scanning Position Weight Matrices (PWM) using FIMO v5.4.1,^14^ a part of the MEME-Suite.^15^ The search was carried out using the JASPAR CORE plants database.^16^ Sites with a *p*-value ≤ 1 × 10^−4^ were considered, and the resulting sites were evaluated using False Discovery Rate (FDR) control, retaining only those with *q* ≤ 0.05.

#### Annotation and visualization

The FIMO output file was processed using a UNIX script that was based on awk|sort. The sites were grouped by sequence, retaining their complete positional data. A non-redundant list of motifs per upstream region was generated. Data analysis and visualization were performed in R v4.3.0. The integration of annotation metadata (TF Family and positional coordinates) was carried out with TFBSTools v1.38.0^17^ and the JASPAR2024 database. The positional distribution of TFBS was visualized with ggplot2 v3.4.2, using coordinates relative to the ATG across the putative promoter range (607–1088 bp).

### Single-cell RNA-seq data retrieval and analysis

Single-cell RNA-seq expression data for MKK genes were retrieved from the publicly available *Arabidopsis* root single-cell atlas^18^; GEO accession GSE123013). Raw counts matrices were processed using Seurat v4 in R.^19^ Data were normalized, scaled, and clustered using standard workflows. Cell types were assigned based on known root cell type marker genes.

## Results

### The promoters of 10 genes encoding MAP kinase kinases in *A. thaliana* were used to construct *UidA* reporter lines

To gain insight into the regulatory mechanism that operates in mitogen-activated protein MAP kinase modules, the promoters of the 10 MKKs genes annotated in *A. thaliana* genome were predicted *in silico* using *The Arabidopsis Information Resource* and *Plant Prom DB* databases (https://www.arabidopsis.org) and cloned with the oligonucleotides mentioned in the materials and methods. Each promoter was fused to the open reading frame (ORF) of the *UidA* reporter with the destiny binary vector pMDC163 (Figure 1a). Thus, 10 constructs formed by a MKK promoter (978 bp for *pMKK1*; 769 bp for *pMKK2*; 1088 bp for *pMKK3*; 851 bp for *pMKK4*; 611 bp for *pMKK5*; 836 bp for *pMKK6*; 950 bp for *pMKK7*; 607 bp for *pMKK8*; 1012 bp for *pMKK9*; 609 bp for *pMKK10*), the *UidA* ORF, and a hygromycin resistance cassette were introduced to *A. thaliana* plants (Figure 1b). The expression patterns observed across all ten reporter lines were summarized and compared with publicly available single-cell transcriptomic atlas data in Table 1. ^18^ The *pMKK2:UidA*, *pMKK3:UidA*, *pMKK4:UidA*, and *pMKK9:UidA* expression was consistent with the atlas records, while the *pMKK1:UidA*, *pMKK5:UidA*, *pMKK6:UidA*, and *pMKK7:UidA* expression partially coincided. The comparison of *pMKK8:UidA* and *pMKK10:UidA* was not performed because the atlas does not have records of MKK8 and MKK10 expression.

**Table 1.** Summary of expression patterns of the 10 MKK genes in *Arabidopsis thaliana* seedlings.

Group	Gen	Primary root tip (PRT)		LRFZ	CZS	Cotyledon	Temporal pattern	Cell types	Single-cell DD
		Stele/vascular	Meristem/QC	Vascular				(Single-cell atlas)*	
A	MKK1	↑ with age	—	Yes	Yes	↑ apex and vascular	Increases with DAG	Epidermis, cortex	Partial
	MKK2	↑ with age	—	Yes	Yes	↑ apex and vascular	Similar to MKK1	All cell types	Consistent
	MKK6	↑ with age	—	Yes	Yes	Entire cotyledon from 2 DAG	Increase with DAG	Pericycle	Partial
B	MKK3	Yes	QC + columella	↑ with age	↑ with age	↑ with age	QC/columella ↓, vascular ↑	Cortex, vascular	Consistent
C	MKK4	Strong	Stem cell niche	↑ with age	↑ until 6 DAG	↑ from 2 DAG	Broad, increase with DAG	Cortex/endodermis, vascular	Consistent
	MKK5	Faint	Faint	↑ with age	Absent until 10 DAG	↑ apex	More restricted than MKK4	Broad, except meristem and epidermis	Partial
D	MKK7	Yes	—	Yes	Yes	↑ with age	Increases with DAG	Very low, generalized	Partial
	MKK8	Yes	—	Yes	Yes	↑ with age	Similar to MKK7	not detected in the atlas	n/a
	MKK9	Yes	—	Yes	Yes	↑ with age	Similar to MKK7/8	Broad, except some clusters	Consistent
	MKK10	—	—	—	Small areas	↓ dramatically at 4 DAG, ↑ later	Most restricted pattern	not detected in the atlas	n/a

Expression data from *pMKK::UidA* reporter lines observed at 2, 4, 6, 8, and 10 days after germination (DAG) are compared with single-cell RNA-seq data retrieved from a publicly available Arabidopsis root atlas. PRT, primary root tip; LRFZ, lateral root formation zone; CZS, central zone of the shoot; QC, quiescent center; n/a, gene not detected in the single-cell atlas.

Yes = expression detected; ↑ = increases with age; ↓ = decrease expression; Faint = weak/restricted expression; — = absent; n/a = gene not detected in atlas.

*Zhang et al.^18^ GEO accession GSE123013.

#### Expression patterns of the group A *MKKs*

**Figure 2.**
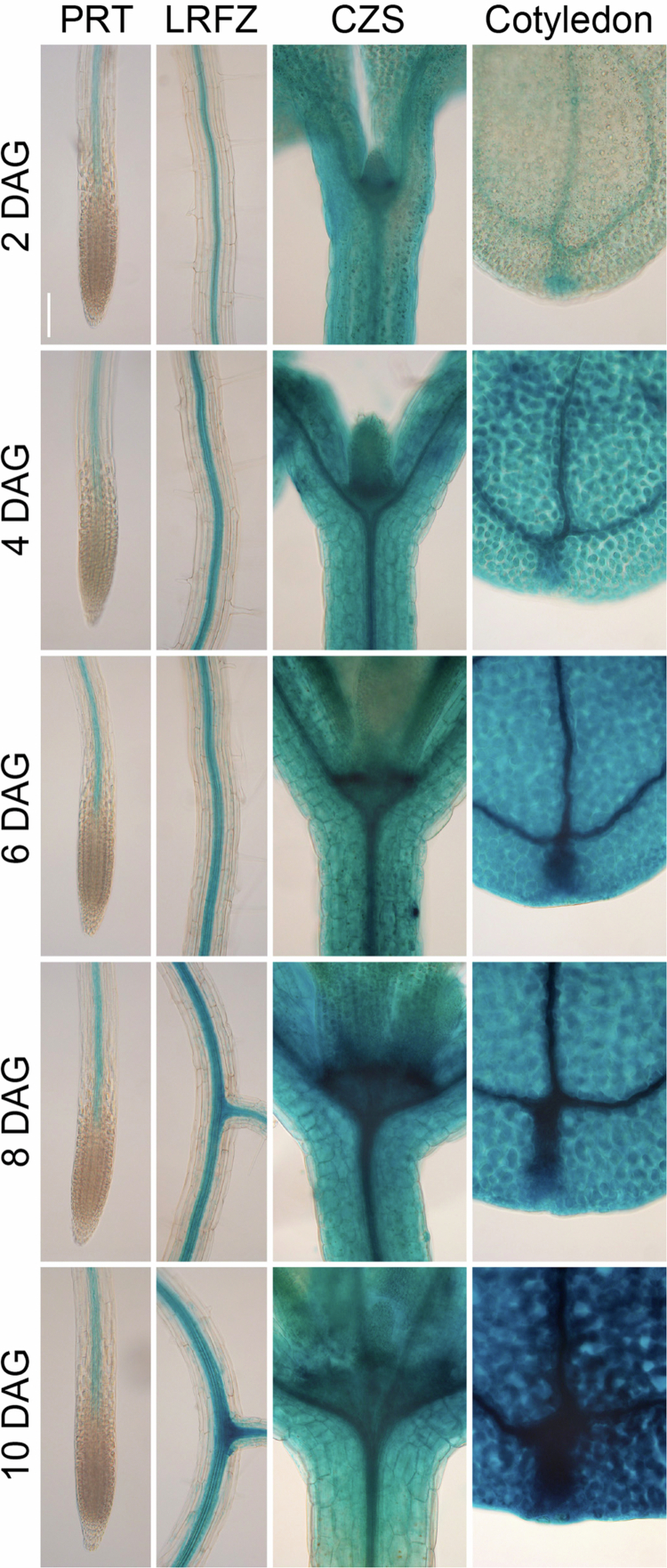
Expression pattern of *pMKK1:UidA* in wild-type seedlings during the postembryonic stage. Seeds carrying the *pMKK1:UidA* construct were sown and seedlings of 2, 4, 6, 8, and 10 d after germination (DAG) were incubated with X-Gluc for the β-glucuronidase Assay. Micrographs of the primary root tip (PRT), the lateral-root formation zone (LRFZ), the central zone of the shoot (CZS), and the cotyledon were taken. Each image is representative of 12 individuals analyzed, and the experiment was repeated twice. Scale bar = 100 µm.

Seedlings carrying the *pMKK1:UidA, pMKK2:UidA,* and *pMKK6:UidA* constructions were sown on MS 0.2X media during two, four, six, eight, and ten days after germination (DAG), and they were evaluated by the β-glucuronidase staining assay. Then, primary root tip (PRT), lateral-root formation zone (LRFZ), center zone of shoot (CZS), and cotyledon were photographed. The PRT and LRFZ micrographs showed that *MKK1* was slightly expressed in the stele at two DAG, whose intensity gradually increased according to the seedling age increment. However, *MKK1* was not found in the meristematic zone or other tissue layer at the root (Figure 2). *MKK1* expression occurred throughout CZS and its expression was gradually magnified in the vasculature as the seedlings were older. Cotyledon of two DAG had a slight expression at the apex and vasculature; however, the expression occurred throughout the structure from four DAG and was gradually magnified in the apex and vasculature during 4, 6, 8, and 10 DAG (Figure 2). The expression patterns of *pMKK2:UidA* (Figure S1) were similar to those found in *pMKK1:UidA* at the same times and tissues. The *pMKK6:UidA* was expressed similarly to its two group mates, with the difference that it was observed in the entire cotyledon from 2 DAG (Figure S2). The expression pattern of *pMKK1:UidA* (Figure 2) and that of *pMKK2:UidA* (Figure S1) were similar in the tissues and times analyzed. These results support functional redundancy between the two MAP kinases. The expression and consequent synthesis of protein at a specific time and place further supports the hypothesis that both kinases participate in the same biological processes.

#### Expression patterns of the *MKK* from the group B

**Figure 3.**
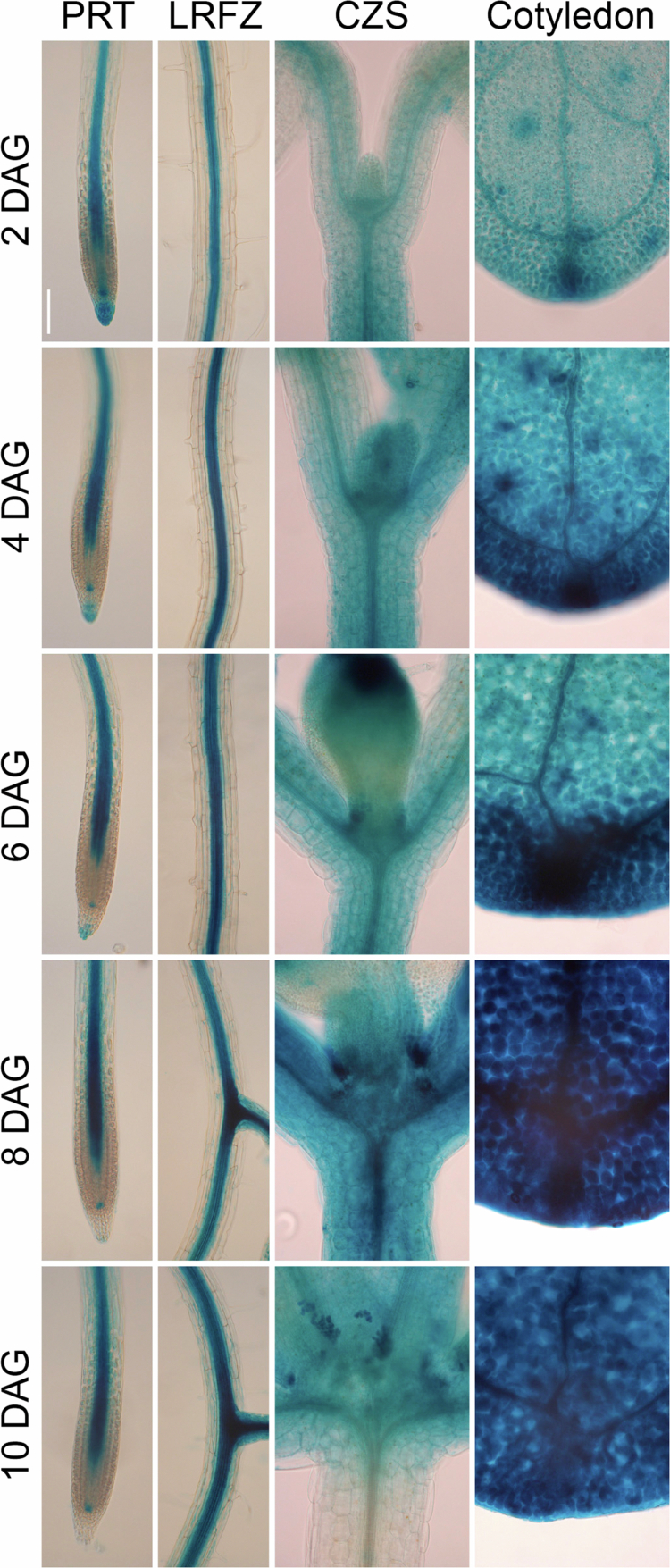
Expression pattern of *pMKK3:UidA* in wild-type seedlings during the postembryonic stage. Seeds carrying the *pMKK3:UidA* construct were sown, and seedlings at 2, 4, 6, 8, and 10 d after germination (DAG) were incubated with X-Gluc for the β-glucuronidase assay. Micrographs of the primary root tip (PRT), the lateral-root formation zone (LRFZ), the central zone of the shoot (CZS), and the cotyledon were taken. Each image is representative of 12 individuals analyzed, and the experiment was repeated twice. Scale bar = 100 µm.

The β-glucuronidase assay in seedlings carrying the *pMKK3:UidA* construction showed *MKK3* expression in the stele, quiescent center, and columella of PRT during 2, 4, 6, 8, and 10 DAG. *MKK3* expression gradually decreased in the quiescent center and columella as the seedlings were older. Inversely, the reporter expression was higher in the vascular cylinder of both PRT and the lateral root formation zone, in accordance with the increase in the DAG (Figure 3). The expression of MKK3 in the CSZ and through cotyledons was progressively increased as the seedling grew (Figure 3). The *pMKK3:UidA* expression in the quiescent center, the root apical meristem and the columella from the first days after germination (DAG), suggests that MKK3 participates in root cell proliferation (Figure 3).

#### Expression patterns of the *MKKs* from the group C

**Figure 4.**
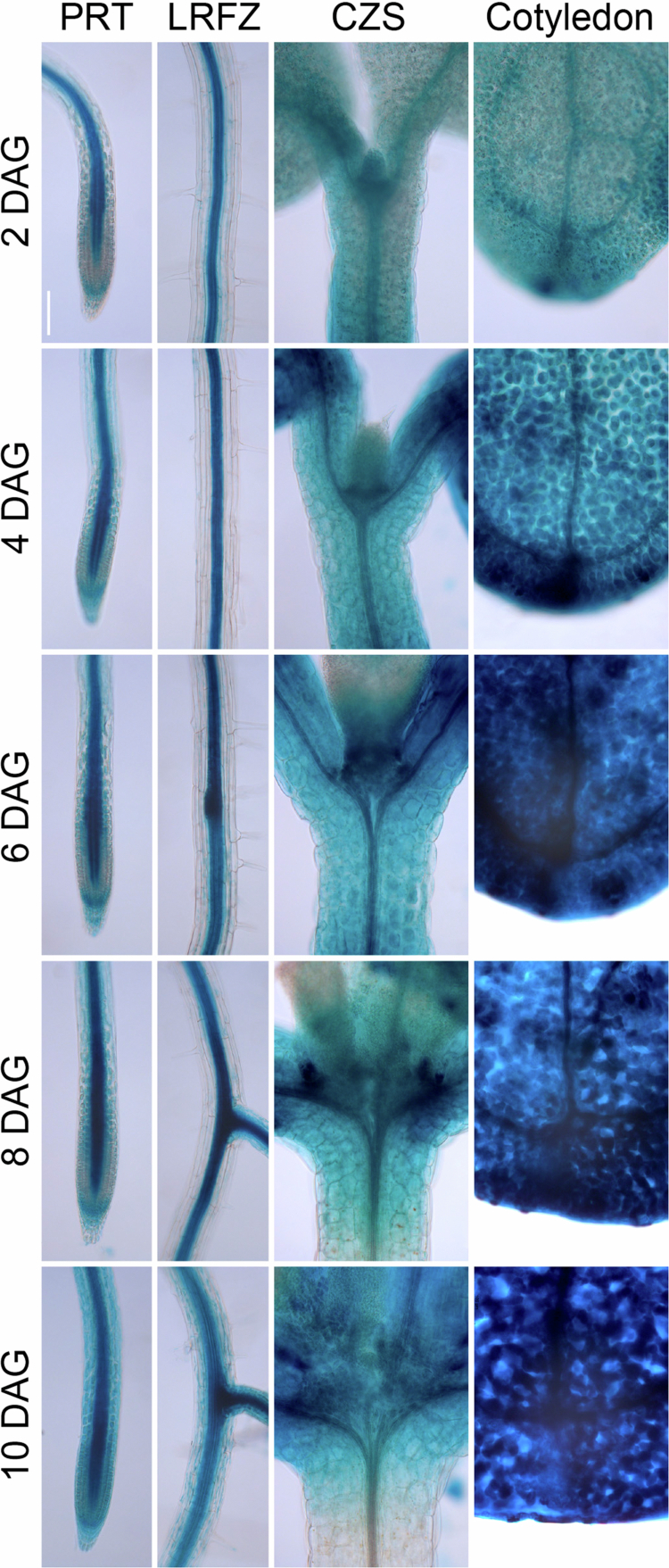
Expression pattern of *pMKK4:UidA* in wild-type seedlings during the postembryonic stage. Seeds carrying the *pMKK4:UidA* construct were sown, and seedlings at 2, 4, 6, 8, and 10 d after germination (DAG) were incubated with X-Gluc for the β-glucuronidase assay. Micrographs of the primary root tip (PRT), the lateral-root formation zone (LRFZ), the central zone of the shoot (CZS), and the cotyledon were taken. Each image is representative of 12 individuals analyzed, and the experiment was repeated twice. Scale bar = 100 µm.

The expression patterns of *pMKK4:UidA* showed a strong expression in the vascular cylinder of the PRT. This MKK is also expressed in the stem cell niche and is gradually spread to all root tissues as plant age increased. In a similar way, *pMKK4:UidA* expression was gradually increased in the vascular cylinder of the lateral root formation zone (Figure 4). In the CZS, the expression of this kinase increased uniformly until 6 DAG and disappeared from the hypocotyl at 8 and 10 DAG. Blue coloration was observed from 2 DAG and increased strongly after 4, 6, 8, and 10 DAG in the cotyledon (Figure 4). The intensity of *pMKK4:UidA* expression as well as its location in the root meristem from 2 DAG, supports MKK4 participation in meristematic processes (Figure 4). In contrast to *pMKK4:UidA*, seedlings carrying the *pMKK5:UidA* showed a faint expression in the vascular cylinder and stem cell niche at 2 DAG, which increased as the seedlings grew, and blue coloration was easily visible at 10 DAG. An age-dependent increment of reporter expression occurred in the vascular cylinder of the LRFZ and the cotyledon apex. The CZS did not show *pMKK5:UidA* expression until 10 DAG (Figure S3).

#### The *pMKK7:UidA*, *pMKK8:UidA*, *pMKK9:UidA*, and *pMKK10:UidA* expression in plant tissues

**Figure 5.**
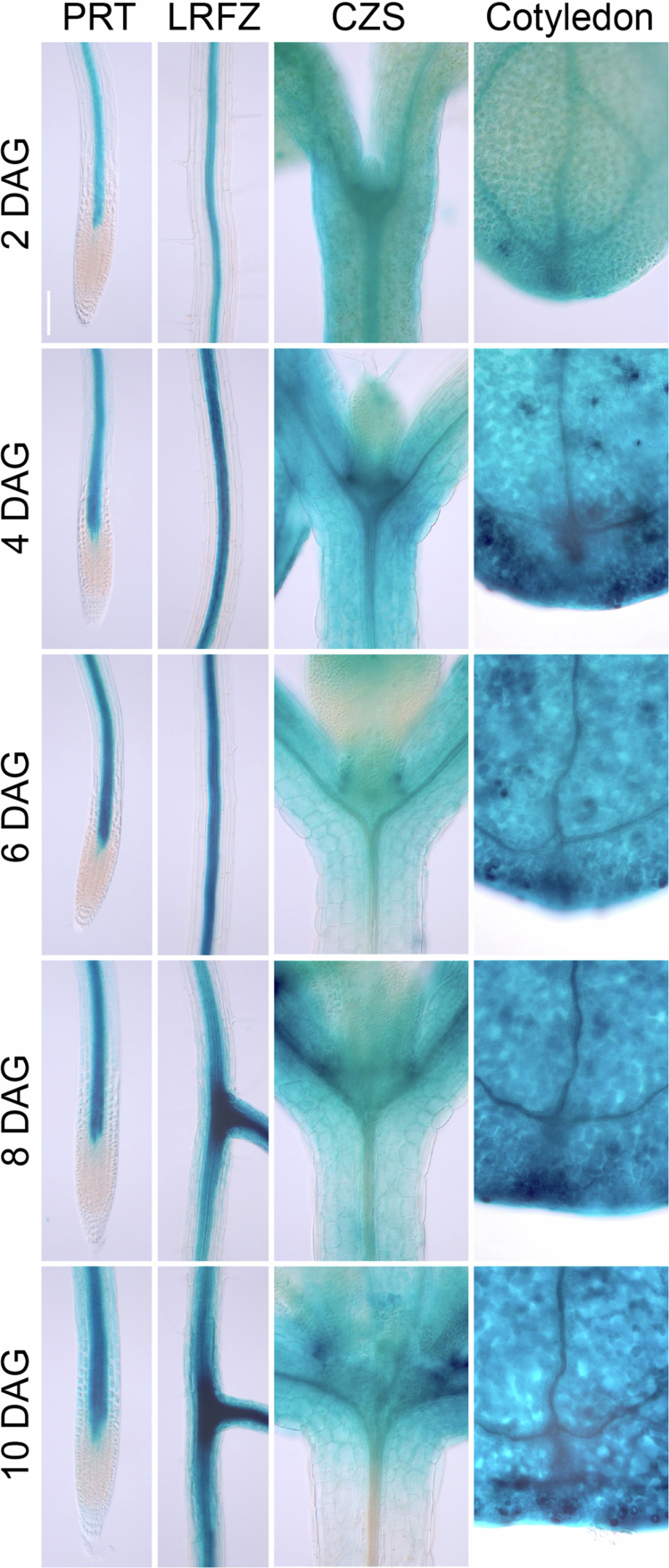
Expression pattern of *pMKK7:UidA* in wild-type seedlings during the postembryonic stage. Seeds carrying the *pMKK7:UidA* construct were sown, and seedlings at 2, 4, 6, 8, and 10 d after germination (DAG) were incubated with X-Gluc for the β-glucuronidase assay. Micrographs of the primary root tip (PRT), the lateral-root formation zone (LRFZ), the central zone of the shoot (CZS), and the cotyledon were taken. Each image is representative of 12 individuals analyzed, and the experiment was repeated twice. Scale bar = 100 µm.

PRT and LRFZ of *pMKK7:UidA* seedlings had β-glucuronidase activity in the vascular cylinder, which was more intense when the seedlings were older (2, 4, and 6 DAG). During 8 and 10 DAG, blue coloration spread to other root tissues but not to the meristem. In CZS, the reporter expression was gradually relocated disappearing from the hypocotyl from 6 DAG and was homogeneously sustained from 4 DAG in the cotyledon (Figure 5). *pMKK8:UidA* and *pMKK9:UidA* had a similar expression pattern to *pMKK7:UidA* but with some differences. The β-glucuronidase activity was less intense in the vascular cylinder of *pMKK8:UidA*during all days of the analysis, and occurred with great intensity from 2 DAG in the cotyledon (Figure S4). The *pMKK9:UidA* expression disappeared in the hypocotyl from 4 DAG (Figure S5). For its part, *pMKK10:UidA* was the member with the most different expression pattern. The blue coloration was not observed in the root (PRT and LRFZ) during the time analyzed (2–10 DAG), and CZS had small areas of expression, mainly in the hypocotyl. The cotyledon had a homogeneous expression at 2 DAG, which decreased dramatically at 4 DAG (located only in the vein) and gradually increased at the cotyledon tip during 6, 8, and 10 DAG (Figure S6).

#### Architecture of *cis*-regulatory motifs in MKK gene putative promoters

To determine whether the observed expression patterns in the reporter lines coincided with shared or specific regulatory signatures, the 10 upstream regulatory regions of the MKK genes were analyzed to search for potential Transcription Factor Binding Sites (TFBS). These annotations were subsequently used as a basis for the interpretation of the observed expression patterns.

A total of 72 types of *cis*-elements were identified across the 10 analyzed upstream regulatory regions. These motifs are associated with Transcription Factors (TFs) related to growth and development (including the regulation of meristems, the transition to the reproductive phase, organ formation, and tissue differentiation); as well as pathways modulated by environmental signals, light, and hormones (Figure S7; Table S1^20^).

Promoters of group A (*MKK1*, *MKK2*, and *MKK6*) display combinations of *cis*-regulatory elements involved in plant development, light regulation, and hormone signaling. These include DOF (DNA-binding with One Finger) motifs associated with photosynthetic processes and carbon/nitrogen (C/N) metabolism;^21^ GARP/G2-like (GOLDEN2-LIKE, GLK) motifs linked to chloroplast development and the regulation of photosynthesis;^22^ bZIP (basic leucine zipper) motifs involved in light- and hormone-mediated regulation;^23^ as well as several MYB motifs related to developmental processes and secondary metabolism.^24^

*MKK3*, the sole member of group B, harbor *cis*-regulatory motifs associated with photosynthesis, secondary metabolism, and chloroplast development, including GARP/G2-like, MYB, and DOF elements.^21^ ^,^ ^22^ ^,^ ^24^

Promoters of group C (*MKK4* and *MKK5*) contain motifs related to Ca^2+^ signaling, metabolic regulation, and the control of plant development. CAMTA (Calmodulin-binding Transcription Activator), DOF, and MYB motifs were identified and have been described as regulators of gene expression in Ca^2+^-dependent pathways, photosynthesis, and C/N metabolism, respectively.^21^ ^,^ ^24^ ^,^ ^25^

Promoters of group D (*MKK7-MKK10*) are enriched in motifs linked to light response, tissue differentiation, and environmental signaling. These include bZIP, Trihelix, GARP/G2-like, and MYB motifs, which are involved in the regulation of genes associated with photomorphogenesis, leaf development, secondary metabolism, and diverse abiotic stress responses.^22^ ^,^ ^23^ ^,^ ^26^ In particular, the MKK10 promoter stands out within this group by also harboring AHL, IDD, CPP, PLINC, and DOF motifs, suggesting a broader transcriptional regulatory input compared to the other group D members, which is consistent with its distinct expression pattern among them.

#### Promoter-specific motifs of MKKs

**Figure 6.**
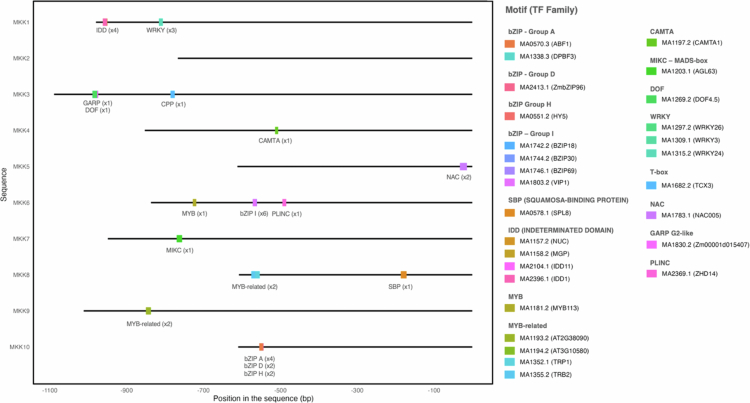
Specific distribution of *cis*-regulatory elements on *MKK* upstream regions. Schematic representation of the *cis*-regulatory elements identified in putative promoters of the *MKK* family, showing their positions relative to the translation start site (ATG). In cases where multiple binding sites belonging to the same transcription factor family are overlapping or located in close proximity, they are displayed as a single colored-rectangle; the number indicated in parentheses denotes the total number of motifs detected in that region. The identity of the transcription factor families is indicated below each colored rectangle in the sequence. TF = transcription factor.

In addition to the conserved patterns within each group, motifs whose presence is restricted to a single promoter were identified. Nine of the ten upstream regulatory sequences analyzed contained exclusive *cis-*regulatory motifs (Figure 6; Table 2).

**Table 2.** Excusive *cis*-regulatory elements on upstream sequences of *MKKs*.

	Sequence	Matrix_ID (JASPAR)	Class	Family	Name	Start	End
Group A	MKK1	MA1157.2	C2H2 zinc finger factors	IDD(INDETERMINATE DOMAIN)	NUC(NUTCRACKER)	−29	−18
		MA1158.2			MGP(MAGPIE)	−30	−18
		MA2104.1			IDD11	−29	−18
		MA2396.1			IDD1	−29	−18
		MA1297.2	GCM domain factors(Glia cell missing) domain factors	WRKY	WRKY26	−174	−164
		MA1309.1			WRKY3	−174	−164
		MA1315.2			WRKY24	−174	−164
	MKK6	MA1181.2	Tryptophan cluster factors	MYB	MYB113	−118	−109
		MA1742.2	bZIP(basic Leucin Zipper domain)	Group I	bZIP18	−274	−265
		MA1744.2			bZIP30	−274	−266
		MA1746.1			bZIP69	−275	−265
		MA1803.2			VIP1(VIRE2-INTERACTING PROTEIN 1)	−274	−265
		MA2369.1	Homeo domain factors	PLINC(PH domain-like inserted in the N-terminal of a homeodomain transcription factor)	ZHD14(Zinc-finger homeodomain protein 14)	−352	−342
Group B	MKK3	MA1269.2	Other C4 zinc finger-type factors	DOF domain(DNA-binding with One Finger domain)	DOF4.5(DNA-BINDING WITH ONE FINGER 4.5)	−112	−100
		MA1682.2	CPP(Cysteine-rich Polycomb-like Protein)		TCX3(TSO1-like CXC domain-containing protein 3)	−314	−303
		MA1830.2	Tryptophan cluster factors	GARP/G2-like(GOLDEN2-LIKE (GLK))	Zm00001d015407	−115	−100
Group C	MKK4	MA1197.2	GCM domain factors(Glia cell missing) domain factors	CAMTA(Calmodulin-binding Transcription Activator)	CAMTA1(Calmodulin-binding Transcription Activator 1)	−347	−339
	MKK5	MA1783.1		NAC(NAM, ATAF1/2, CUC2 domain)	NAC005NAC TRANSCRIPTION FACTOR 29	−597	−579
Group D	MKK7	MA1203.1	MADS box factors	MIKC-type Type II(MADS Intervening, Keratin-like (K), C-terminal)	AGL63(AGAMOUS-LIKE MADS-BOX PROTEIN AGL21)	−193	−179
	MKK8	MA0578.1	C3H(C),C2HC zinc finger factors	SBP(SQUAMOSA Promoter-Binding Protein)	SPL8(SQUAMOSA PROMOTER-BINDING-LIKE PROTEIN 8)	−436	−421
		MA1352.1	Tryptophan cluster factors	MYB-related	TRP1(TELOMERE REPEAT BINDING PROTEIN 1)	−54	−34
		MA1355.2			TRB2(TELOMERE REPEAT BINDING FACTOR 2)	−42	−32
	MKK9	MA1193.2	Tryptophan cluster factors	MYB-related	AT2G38090	−174	−162
		MA1194.2			AT3G10580	−175	−162
	MKK10	MA0551.2	bZIP(basic Leucin Zipper domain)	Group H	HY5(ELONGATED HYPOCOTYL 5)	−66	−55
		MA0570.3		Group A	ABF1(ABSCISIC ACID RESPONSIVE ELEMENT-BINDING FACTOR 1)	−66	−57
		MA1338.3			DPBF3(ABA-RESPONSIVE ELEMENT BINDING PROTEIN 3)	−66	−56
		MA2413.1		Group D	ZmbZIP96	−66	−56

List of exclusive *cis*-regulatory elements identified in the promoters of each *MKK*, indicating the ID in JASPAR, the class and family of the associated transcription factor, the identified motif, and its relative position with respect to the translation start site (ATG).

In group A, the *MKK1* promoter contains IDD (INDETERMINATE DOMAIN) motifs, which are associated with the regulation of vegetative development and leaf architecture,^27^ as well as WRKY motifs linked to defense and stress responses.^28^ The MKK6 promoter harbors a MYB motif associated with metabolic regulation and hormone responses,^24^ together with three group I bZIP motifs related to physiological adjustment under stress conditions.^29^

In group B, the *MKK3* promoter contains a DOF-type motif involved in the regulation of processes such as hormone responses, C/N metabolism, and photosynthesis.^21^ It also includes a CPP (Cysteine-rich Polycomb-like Protein) motif associated with the control of cell division and the development of reproductive tissues,^30^ as well as a GARP/G2-like motif related to chloroplast biogenesis and the regulation of photosynthesis-associated processes.^22^

In group C, the *MKK4* promoter contains a CAMTA motif associated with the integration of Ca^2+^-dependent signaling and the control of responses to multiple types of stress.^25^ In contrast, the *MKK5* promoter harbors a NAC (NAM, ATAF1/2, CUC2 domain) motif involved in the regulation of diverse developmental processes, including senescence and responses to different types of stress.^31^

Finally, among the members of group D, the promoters of *MKK7-MKK10* display combinations of *cis*-regulatory elements associated with developmental processes and responses to environmental cues. The *MKK7-MKK9* promoters contain MIKC (MADS Intervening, Keratin-like [K], C-terminal)-type motifs related to the regulation of developmental programs, including organ identity and reproductive phase transitions.^32^ These three promoters also harbor a PLINC (PH-like domain inserted in the N-terminal of a homeodomain transcription factor) motifs, whose corresponding TFs have been reported to regulate meristem and vegetative organ development through interactions with ZF-HD domains.^33^ Additionally, the promoters of *MKK8* and *MKK9* contain MYB-related motifs associated with metabolic regulation and responses to different types of stress.^24^ Trihelix motifs were identified in the promoters of *MKK9* and *MKK10*; these motifs participate in light responses, leaf and seed development, and tolerance to various abiotic stresses.^26^ The MKK10 promoter also contains bZIP motifs belonging to groups A, D, and H, which are related to ABA-dependent signaling, the regulation of defense mechanisms, and photomorphogenesis, respectively.^23^ ^,^ ^34^ ^,^ ^35^

## Discussion

### The 10 reporter lines of MAP kinase kinases

Transcription represents the transfer of genetic information from DNA to the short-lived messenger RNA, usually with subsequent production of a protein that will carry out a specific function. Although all cells in an organism contain essentially the same DNA, they can function in very diverse ways because of qualitative and quantitative differences in their gene expression. Thus, the control of gene expression is at the heart of differentiation and development.^36^ For the above reasons, it is essential to know where and when the *MKK*s expression occurs in plant tissues. To date, there are several studies on the role of MAPKKs in plant function, however, information is scarce for some members of this type of kinases. Expression assays under different stimuli and growth conditions using the *UidA* constructions generated in this study (Figure 1) will reinforce previous findings and will generate new knowledge about the MKK functioning in plants.

### Group A of *A. thaliana MKKs*

Gao and coworkers^37^ reported that MKK1 and MKK2 negatively regulate innate immune responses in plants. Using mutants affected in the genes encoding these kinases, the study showed that the *mkk1 mkk2* double mutant is resistant to pathogens and exhibits a phenotype distinct from the wild-type. However, *mkk1* and *mkk2* single mutants were not resistant to pathogens, and their phenotype was similar to the wild-type. Other studies have shown that the MKK1/2-MPK4 module mediates tolerance to cold and osmotic stress,^37^ ^,^ ^38^ in addition to innate immunity responses against different pathogens and ROS.^3^ ^,^ ^39^ The MKK1-MPK6 module is also implicated in ABA-dependent *CAT1* expression and drought/salt responses,^40^ as well as sugar signaling during seed germination.^41^ Therefore, the shared vascular localization of MKK1 and MKK2 places both kinases in a tissue where diverse stress signals converge, which is consistent with their proposed role as integrators of abiotic and biotic responses.^37^ ^,^ ^39^ Consistent with this, the MKK1 promoter contains IDD motifs (IDD1, IDD11, NUC, MGP), and WRKY motifs (WRKY3, WRKY24, WRKY26), which participate in the regulation of root growth and transcriptional activity in vascular tissues under defense-related stimuli,^27^ ^,^ ^28^ in agreement with the *pMKK1:UidA* expression observed in the vascular cylinder and cotyledons (Figure 2). MKK2 shares a similar promoter composition and expression pattern with MKK1, further supporting their functional redundancy.

Regarding MKK6, the other kinase of the group A, RT-PCR assays were performed on various plant zones, and *MKK6* expression in roots and shoots was reported^42^; however, this strategy did not allow establishing the expression in specific tissues. The age-dependent increase in *pMKK6:UidA* expression in the vascular tissue of seedlings (Figure S2) indicates the relevance of MKK6 for proper plant development and is consistent with the dwarfism phenotype observed in the *anq1* mutant, which may reflect the contribution of *MKK6* activity in multiple tissues during seedling growth.^43^ MKK6 has also been reported to control cell cycle progression and cytokinesis by interacting with MPK4/11, reinforcing its role in growth-related developmental processes.^44^ In accordance with this, the MKK6 promoter contains MYB, group I bZIP, and PLINC motifs, which are associated with metabolism in young foliar tissues,^24^ physiological adjustment under stress conditions,^29^ and the regulation of vegetative development,^33^ respectively. At the level of sequence-specific elements, MKK6 contains MYB113, ZHD14, and several group I bZIP motifs associated with foliar metabolism, diverse physiological adjustments in response to stress, and vegetative development,^24^ ^,^ ^29^ ^,^ ^33^ which is consistent with the activity of *pMKK6:UidA* in cotyledons and its progressive intensification toward the vascular cylinder (Figure S2).

### *A. thaliana MKKs* of group B

MKK3 has been implicated during the responses of *Arabidopsis* to jasmonic acid (JA) in particularly, negatively modulates primary root growth inhibition by JA.^7^ The above is consistent with the negative regulation of MKK3 in JA-dependent root growth. Supporting this, the MKK3 promoter contains motifs of the AHL, IDD, DOF, and GARP/G2-like families. CPP motifs regulate cell division and the development of reproductive tissues,^30^ whereas DOF and GARP/G2-like motifs are associated with metabolism and the maturation of photosynthetic tissues.^21^ ^,^ ^22^ At the level of sequence-specific elements, the MKK3 promoter contains a DOF4.5 motif involved in hormone responses, C/N metabolism, and photosynthesis;^21^ and a GARP/G2-like motif (Zm00001d015407) related to vascular tissue differentiation and chloroplast biogenesis.^45^ Taken together, the *MKK3:UidA* expression in the quiescent center, columella, and vascular cylinder, reflects the function of these motif families in tissues requiring active proliferation and cellular differentiation (Figure 3).

### *A. thaliana MKKs* of group C

The double *mkk4 mkk5* mutant has short roots, whereas *mkk4* and *mkk5* single mutants are like wild-type, revealing that MKK4 and MKK5 redundantly function in the maintenance of the root meristem.^8^ ^,^ ^46^ The early vascular and stem cell expression correlates with the known roles of MKK4 in developmental and immune signaling, particularly via MPK3/6 cascades.^47^ ^,^ ^48^ Moreover, the vascular-localized expression is consistent with the function of MKK4 in defense signal propagation through vascular tissues,^49^ and the progressive expression observed in cotyledons also aligns with its proposed role in preparing photosynthetic tissues for defense responses. This pattern is supported at the promoter level by the presence of BBR/BPC motifs, which regulate the expression of homeotic genes and vascular development,^50^ IDD motifs that modulate leaf architecture and root development,^27^ and CPP motifs that control cell division and the development of reproductive tissues,^30^ and DOF motifs associated with the regulation of photosynthetic processes and tissue maturation.^21^ In particular, the MKK4 promoter contains a sequence-specific CAMTA1 motif, whose role as an integrator of Ca^2+^-dependent signals in transcriptional regulation^25^ is consistent with the expression of *pMKK4:UidA* across multiple root tissues (Figure 4).

In contrast, the expression pattern of *pMKK5:UidA* suggests that the role of MKK5 in plant responses could be secondary to that of MKK4. At the promoter level, Sharif and collaborators^51^ reported that HD-ZIP motifs control meristem and vascular tissue development, which is consistent with the localization of *pMKK5:UidA* expression (Figure S3). Additionally, the MKK5 promoter harbors a sequence-specific NAC005 motif involved in vascular development and growth stage-dependent cellular responses,^31^ which is reflected in the age-dependent increase in the *pMKK5:UidA* signal in the vascular cylinder (Figure S3). The progression of reporter activity for both the MKK4 and MKK5 promoters in cotyledons and roots is consistent with their participation in preparing photosynthetic and vascular tissues for growth and stress responses.^52^

### *A. thaliana MKKs* of group D

*MKKs* from group D are the least studied, in concordance, no functional redundancy has emerged among them. The published information for MKK7 indicates that this kinase participates modulating salicylic acid (SA) during the plant systemic response to pathogens.^9^ Additionally, MKK7 has been involved in lateral root development and auxin signaling, as it forms a module with MPK6 to regulate PIN1-mediated auxin transport.^53^

The *pMKK7:UidA* expression in vascular tissues would explain that MKK7 promotes the SA-dependent systemic response (Figure 5). At the promoter level, MKK7 contains MIKC family motifs AGL63 (related to the regulation of development and tissue differentiation)^32^ as well as PLINC motifs that participate in the regulation of meristem and vegetative organ development through interactions with ZF-HD domains,^33^ both of which are consistent with the progressive expansion of *pMKK7:UidA* expression to multiple root tissues as development proceeds (Figure 5).

The phylogenetic closeness between *MKK7* and *MKK8* of *Arabidopsis*,^4^ together with the spatiotemporal similarity of their expression patterns (Figure 5 and Figure S4), suggests a similar role between these two kinases. Consistently, the MKK8 promoter contains PLINC motifs shared with MKK7, as well as sequence-specific elements, including an SBP (SQUAMOSA Promoter-Binding Protein) motif and two MYB-related motifs (TRP1, TRB2), whose involvement in developmental programs of young foliar tissues is consistent with the concentration of *pMKK8:UidA* expression in cotyledons (Figure S4).^24^ ^,^ ^54^

MKK9 displays an expression pattern similar to that of MKK7 and MKK8, however, have shown that the *mkk9* loss-of-function mutant exhibits a broad spectrum of moderate ethylene-insensitive phenotypes, while transgenic plants expressing the constitutively active MKK9 (MKK9a) show constitutive ethylene phenotypes.^10^ This ethylene-related function in whole seedlings correlates well with the broad *pMKK9:UidA* expression observed across many tissues (Figure S5). At the promoter level, MKK9 also contains MYB-related motifs and PLINC elements shared with MKK7 and MKK8, and its reporter activity in cotyledons agrees with the expression patterns described for the MYB family (Figure S5).

MKK10 does not share a similar expression pattern as shown by the other members of the group. It has been proposed that MKK10 function downstream of *phyB* in regulating cotyledon opening in red light when the seedlings emerge from the seeds.^11^ Consistently, *pMKK10:UidA* is slightly expressed in cotyledons at 2 DAG, and this expression decreases dramatically after 4 DAG (Figure S6) these data suggests the participation at early times than those focused in our analysis. However, the reappearance of *pMKK10:UidA* expression at 6 DAG and the increase in its intensity at 8 and 10 DAG, proposes a role for MKK10 in cotyledon functioning in stages later than early plant development. This light-dependent pattern is supported by the promoter composition of MKK10, which contains bZIP motifs from groups H (HY5), A (ABF1, DPBF3), and D (ZmZIP96), related to ABA-dependent signaling, the regulation of defense mechanisms, and photomorphogenesis, respectively^23^ ^,^ ^34^ ^,^ ^35^ Additionally, Trihelix motifs present in the MKK10 promoter participate in light responses, leaf and seed development, and tolerance to various abiotic stresses,^26^ all consistent with the restriction of *pMKK10:UidA* expression to aerial tissues (Figure S6).

Together, the differential phenotypes and responses of the single mutants of MKKs from group D indicate that these kinases do not share function; however, the similar expression patterns of MKK7, MKK8, and MKK9 suggest possible complementary roles.

### Common and expression patterns

The contrasting expression domains observed among MKK family members reinforce that more than one MKK could act receiving and transmitting same upstream stimuli, but also suggest that single MKKS could act alone as shown by the specific expression patterns.

At the promoter level, the bioinformatic analysis of the upstream regulatory regions reveals that this diversity of expression is underpinned by a combination of broadly shared gene-specific *cis*-regulatory motifs. Motifs associated with vascular development, photosynthesis, and stress responses—such as DOF, GARP/G2-like, MYB, and bZIP elements—are present across multiple MKK promoters from all four groups, providing a molecular basis for the overlapping expression patterns observed experimentally. In contrast, exclusive motifs such as IDD and WRKY in MKK1, DOF4.5, CPP, and GARP/G2-like in MKK3, CAMTA in MKK4, NAC005 in MKK5, AGL3 in MKK7, SBP in MKK8, two MYB-related motifs in MKK9 and bZIP groups H, A, and D in MKK10 help explain the unique expression domains characteristics of each kinase. Together, these findings indicate that the regulatory network governing the MKK family operates through a combination of shared and exclusive transcriptional inputs, which likely determines both the redundant and specific biological roles of these kinases in plant development and stress responses.

## Conclusion

The 10 MAPKKs of *A. thaliana* are signaling proteins required from early plant development and whose function occurs in multiple tissues, increasing with respect to time. The high intensity and widespread localization of the expression of some *MKKs*, allows us to infer that the proteins encoded by these genes have an important function in post-embryonic development under control conditions. However, MKKs operate in MAPK modules that are part of more complex signaling pathways. The *UidA* transgenic lines generated in this work could be used to elucidate the MAPK-dependent signaling mechanism that regulates plant growth and development under various growth conditions. Besides, the bioinformatic analysis can be useful as a starting point for future functional studies that later allow understand how plants respond to constantly changing environmental conditions.

## Supplementary Material

Paper MAP2Ks_supplementary.docx Paper MAP2Ks_supplementary.docx

Supplementary material Figure_S5.jpg

Supplementary material Figure_S2.jpg

Supplementary material Figure_S4.jpg

Supplementary material Figure_S6.jpg

Supplementary material Figure_S3.jpg

Supplementary material Figure_S1.jpg

Supplementary material Figure_S7.tiff
