# Supplementary material for: Expression pattern of the 10 mitogen-activated protein kinase kinases (MAPKK) encoded on Arabidopsis thaliana genome
Source: Plant Signal Behav. 2026 Jul 6;21(1):2697589. doi: 10.1080/15592324.2026.2697589 (PMC13348940; doi:10.1080/15592324.2026.2697589)
Supplement: Paper MAP2Ks_supplementary.docx [file KPSB_A_2697589_SM7556.docx]

Expression Pattern of the 10 Mitogen-Activated Protein Kinase Kinases (MAPKK) encoded on *Arabidopsis thaliana* genome

Salvador Barrera-Ortiz^1+^, José Manuel González-Coronel^1+^, Yessica Casales-Tlatilpa^2^, Jesús Salvador López-Bucio^3^, Julián David Camargo-Pacanchique^4^, Ángel Arturo Guevara-García^1^*

^1^Instituto de Biotecnología, Universidad Nacional Autónoma de México, Apartado Postal 510-3, 62250 Cuernavaca, Morelos, México.

^2^Laboratorio de Biología Molecular, Centro de Desarrollo de Productos Bióticos, Instituto Politécnico Nacional, carretera Yautepec-Jojutla Km. 6 Calle CeProBi No 8, Col. San Isidro, 62731. Yautepec, Morelos, México.

^3^Investigador por México SECIHTI-UMSNH, Instituto de Investigaciones Químico Biológicas, Universidad Michoacana de San Nicolás de Hidalgo, Morelia, Michoacán, México.

^4^Pontifica Universidad Javeriana, Ak 7 #40-62, Bogotá, Colombia.

^+^Equal contribution.

***Corresponding author (CONTACT)**: E-mail: arturo.guevara@ibt.unam.mx, telephone: +52 (777) 329-1777 ext. 38134, fax: +52 (777) 313-9988

**Supplementary material**


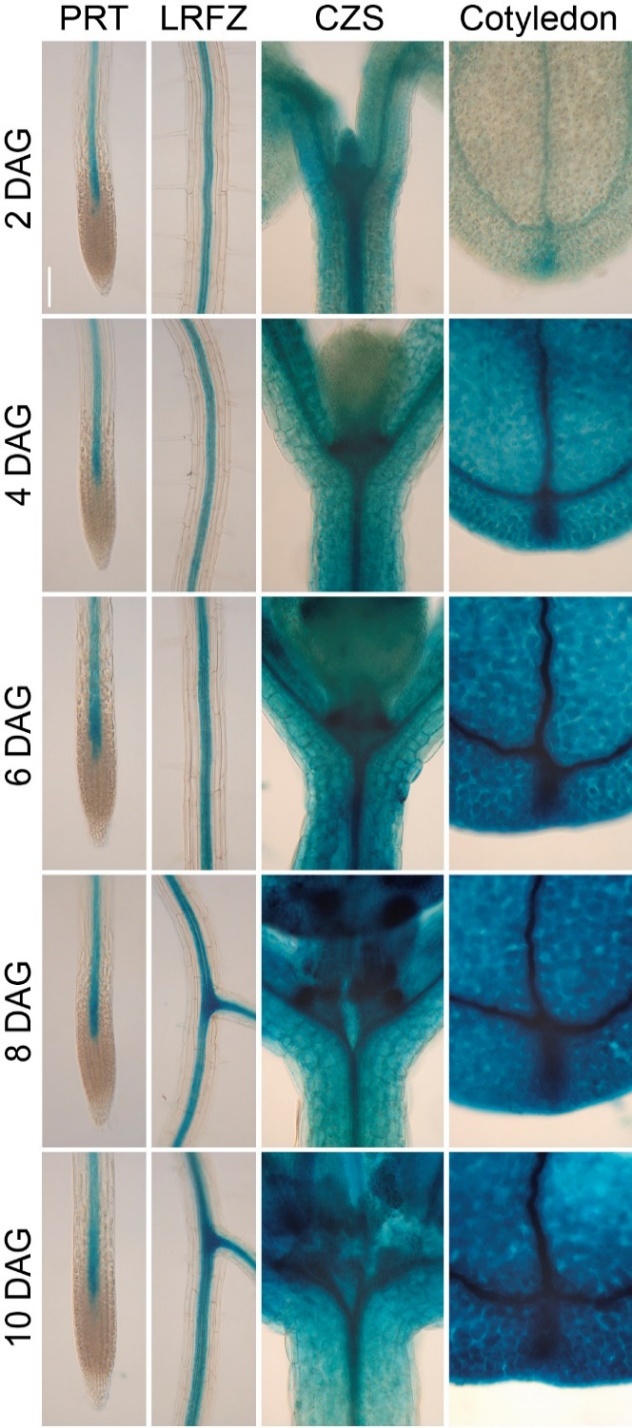


**Figure S1. Expression pattern of *pMKK2::UidA* in wild-type seedlings during the postembryonic stage.** Seeds carrying the *pMKK2::UidA* construct were sown and seedlings of 2, 4, 6, 8, and 10 days after germination (DAG) were incubated with X-Gluc for the β-glucuronidase Assay. Micrographs of the primary root tip (PRT), the lateral-root formation zone (LRFZ), the central zone of ​​the shoot (CZS), and the cotyledon were taken. Each image is representative of 12 individuals analyzed and the experiment was repeated twice. Scale bar = 100 µm.


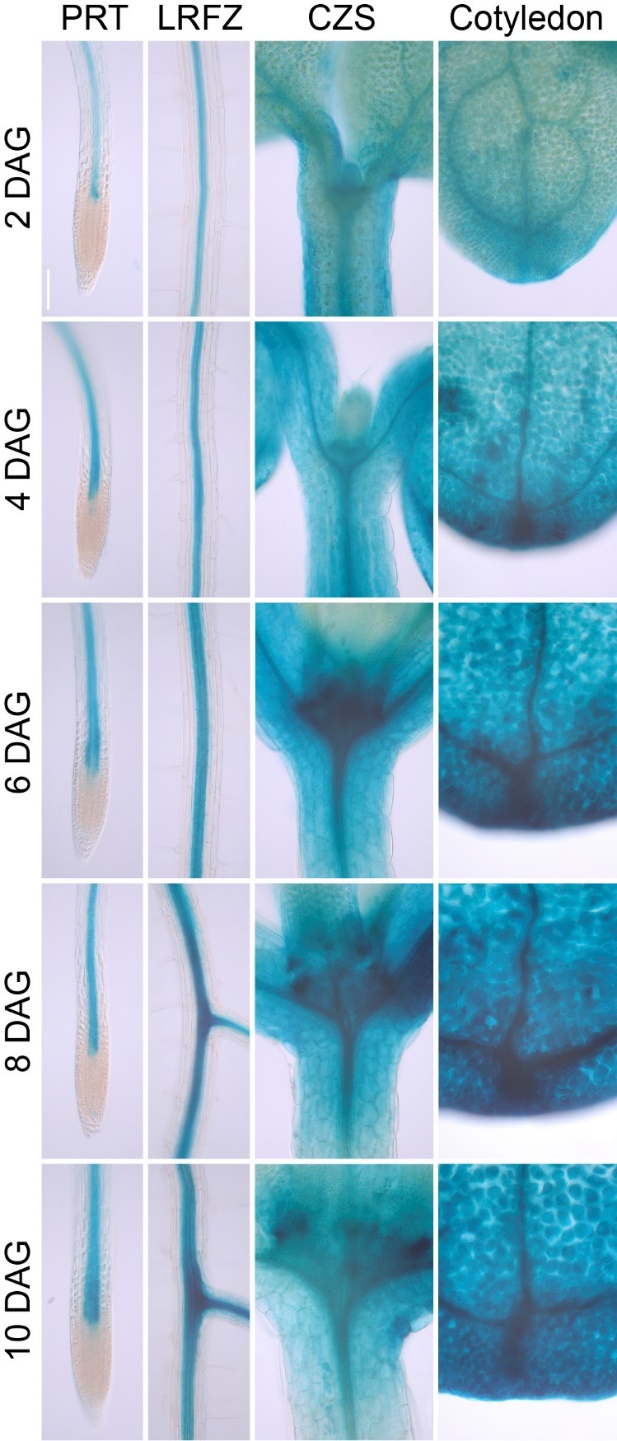


**Figure S2. Expression pattern of *pMKK6::UidA* in wild-type seedlings during the postembryonic stage.** Seeds carrying the *pMKK6::UidA* construct were sown and seedlings of 2, 4, 6, 8, and 10 days after germination (DAG) were incubated with X-Gluc for the β-glucuronidase Assay. Micrographs of the primary root tip (PRT), the lateral-root formation zone (LRFZ), the central zone of ​​the shoot (CZS), and the cotyledon were taken. Each image is representative of 12 individuals analyzed and the experiment was repeated twice. Scale bar = 100 µm.


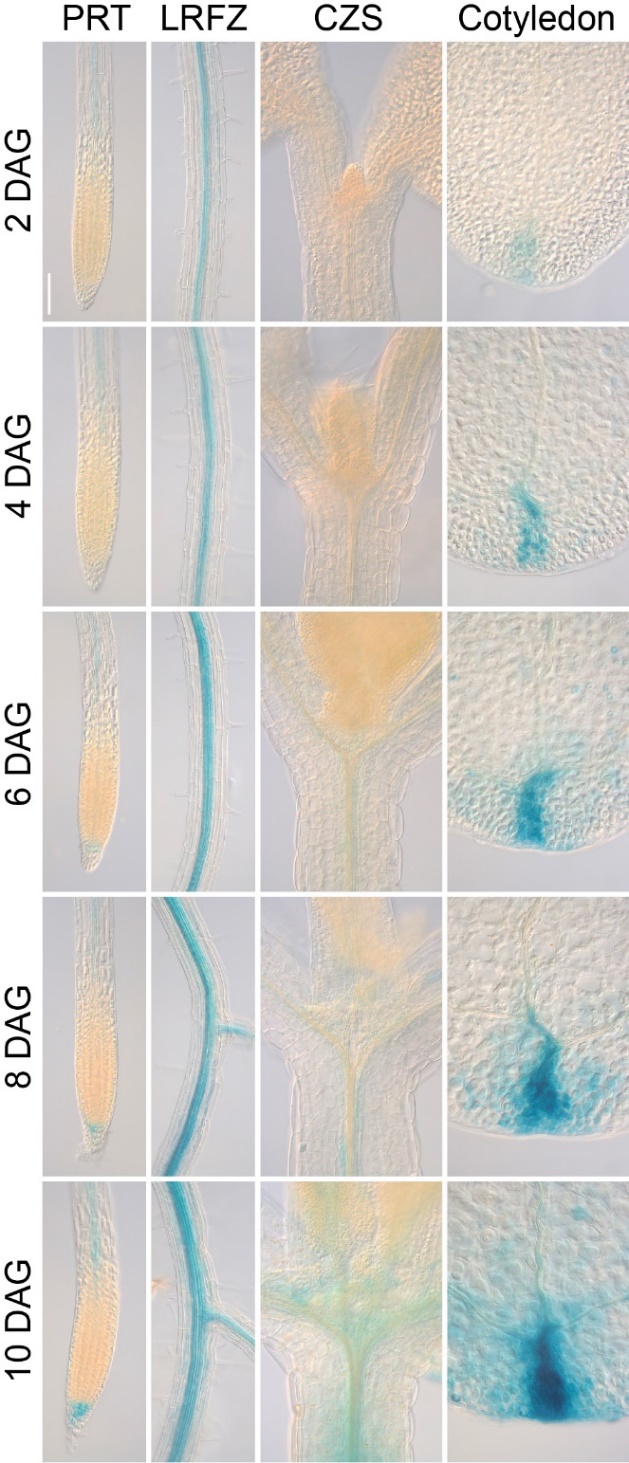


**Figure S3. Expression pattern of *pMKK5::UidA* in wild-type seedlings during the postembryonic stage.** Seeds carrying the *pMKK5::UidA* construct were sown and seedlings of 2, 4, 6, 8, and 10 days after germination (DAG) were incubated with X-Gluc for the β-glucuronidase Assay. Micrographs of the primary root tip (PRT), the lateral-root formation zone (LRFZ), the central zone of ​​the shoot (CZS), and the cotyledon were taken. Each image is representative of 12 individuals analyzed and the experiment was repeated twice. Scale bar = 100 µm.


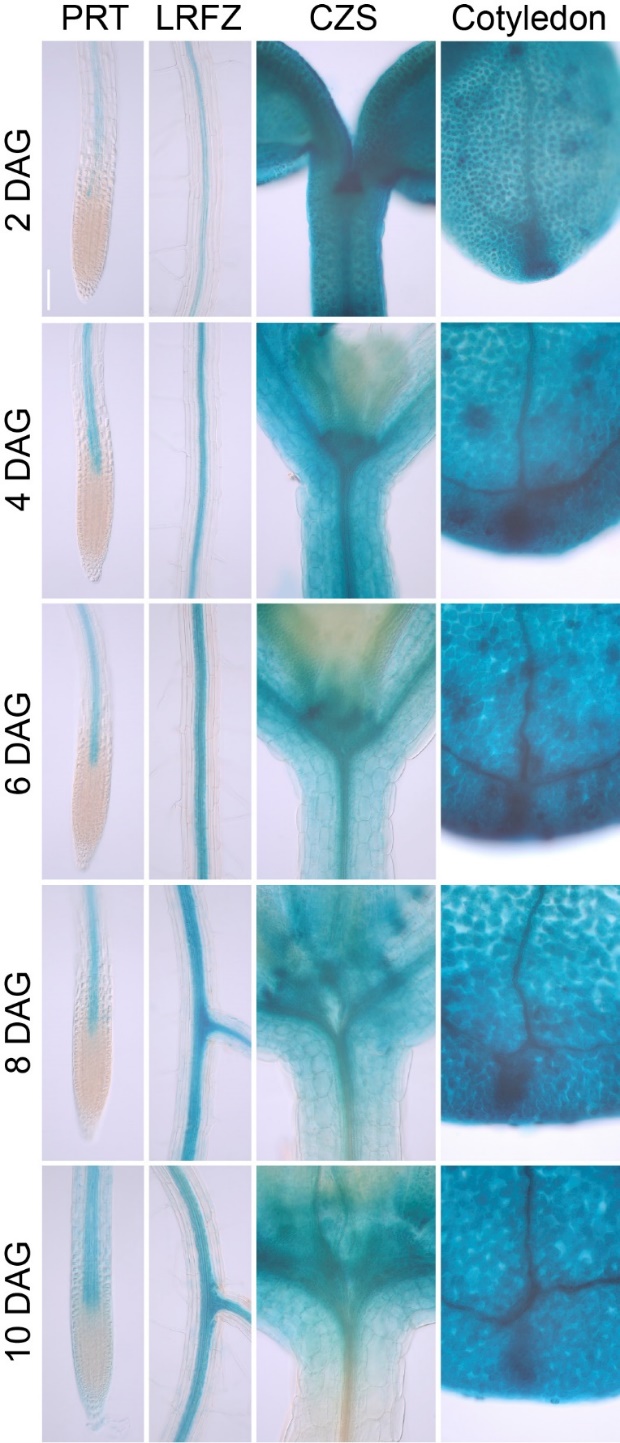


**Figure S4. Expression pattern of *pMKK8::UidA* in wild-type seedlings during the postembryonic stage.** Seeds carrying the *pMKK8::UidA* construct were sown and seedlings of 2, 4, 6, 8, and 10 days after germination (DAG) were incubated with X-Gluc for the β-glucuronidase Assay. Micrographs of the primary root tip (PRT), the lateral-root formation zone (LRFZ), the central zone of ​​the shoot (CZS), and the cotyledon were taken. Each image is representative of 12 individuals analyzed and the experiment was repeated twice. Scale bar = 100 µm.


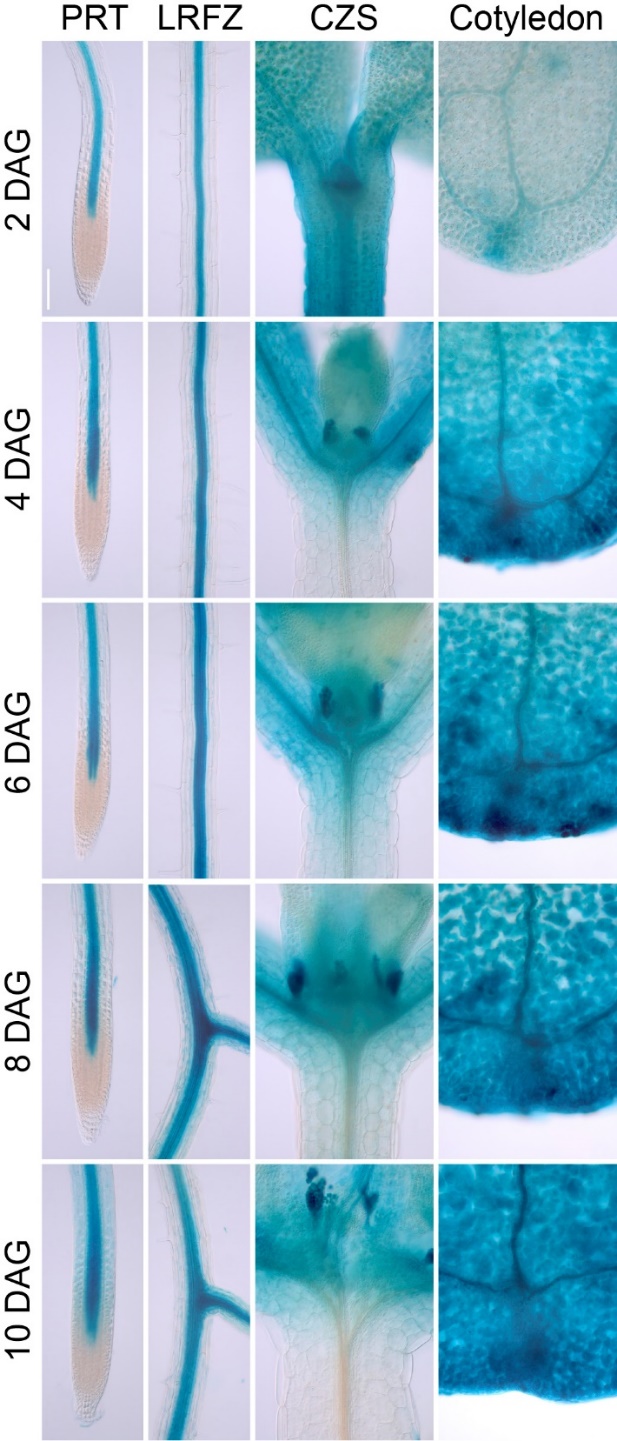


**Figure S5. Expression pattern of *pMKK9::UidA* in wild-type seedlings during the postembryonic stage.** Seeds carrying the *pMKK9::UidA* construct were sown and seedlings of 2, 4, 6, 8, and 10 days after germination (DAG) were incubated with X-Gluc for the β-glucuronidase Assay. Micrographs of the primary root tip (PRT), the lateral-root formation zone (LRFZ), the central zone of ​​the shoot (CZS), and the cotyledon were taken. Each image is representative of 12 individuals analyzed and the experiment was repeated twice. Scale bar = 100 µm.


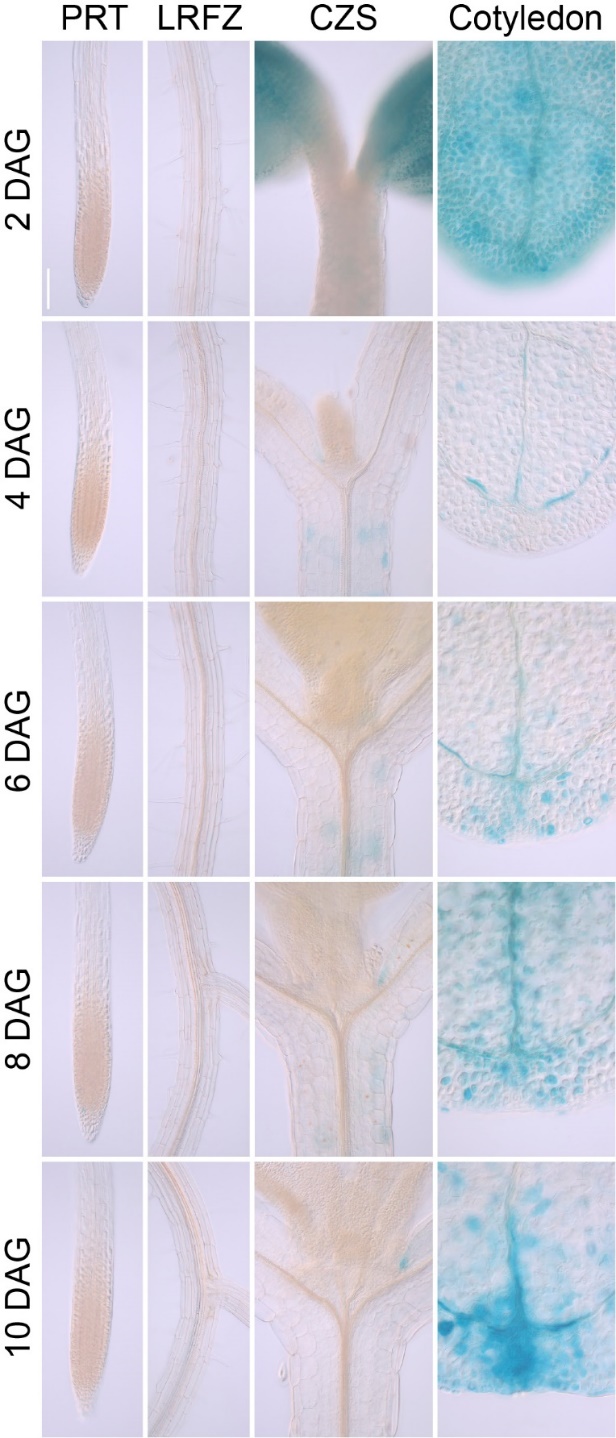


**Figure S6. Expression pattern of *pMKK10::UidA* in wild-type seedlings during the postembryonic stage.** Seeds carrying the *pMKK10::UidA* construct were sown and seedlings of 2, 4, 6, 8, and 10 days after germination (DAG) were incubated with X-Gluc for the β-glucuronidase Assay. Micrographs of the primary root tip (PRT), the lateral-root formation zone (LRFZ), the central zone of ​​the shoot (CZS), and the cotyledon were taken. Each image is representative of 12 individuals analyzed and the experiment was repeated twice. Scale bar = 100 µm.


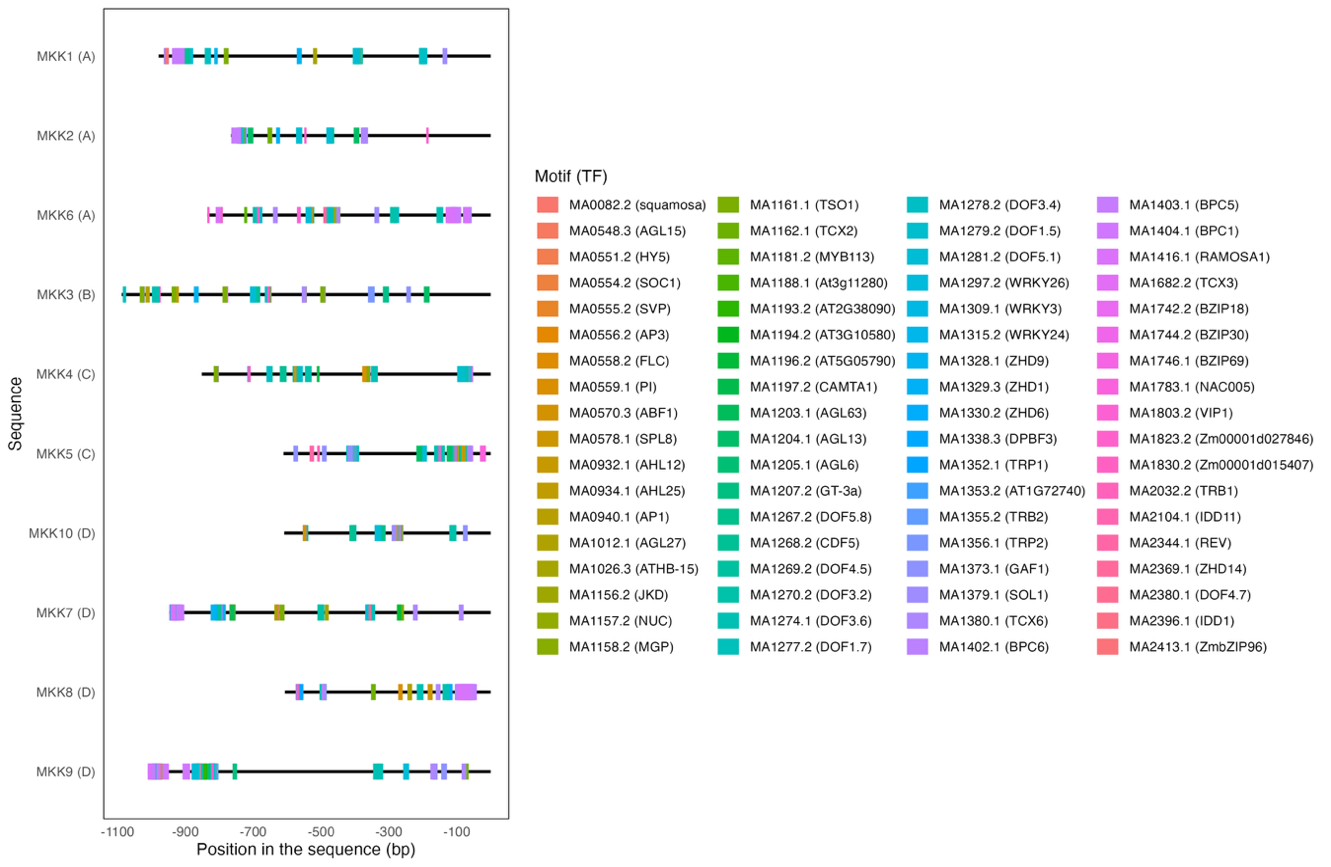


**Figure S7. Distribution and organization of cis elements identified in the putative promoters of the *MKK* genes.** Schematic representation of potential transcription factor binding sites (TFBS) detected by FIMO in the *MKK1-MKK10* promoter sequences, grouped by subfamily (A-D). Colored rectangles indicate individual motifs defined by JASPAR arrays, and their position is shown relative to the translation start site (ATG). The identity of each motif is indicated alongside the colored boxes arranged in columns. TF= transcription factor.

**
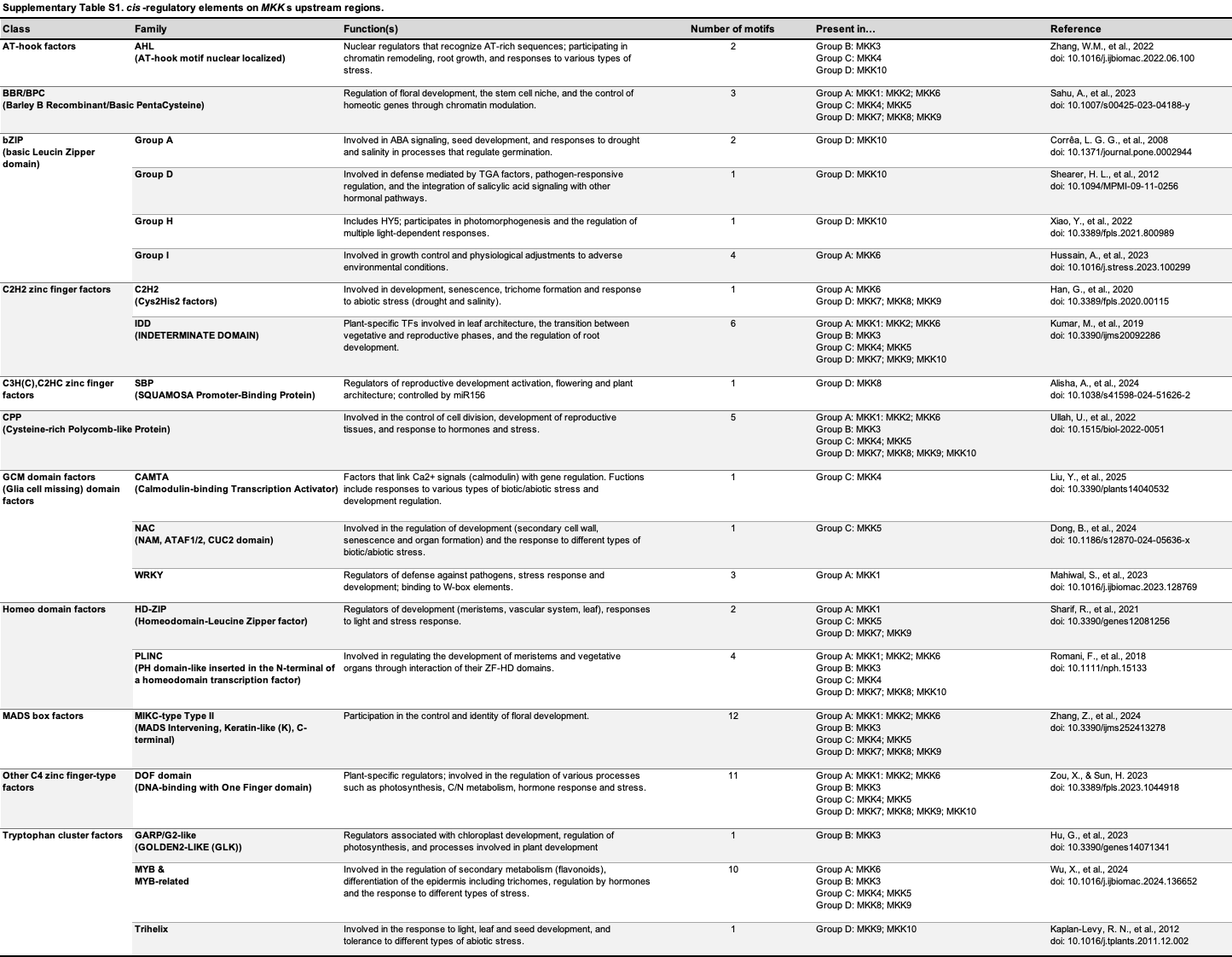
**

**Table S1.*cis*-regulatory elements in putative promoters of the *MKK* genes.** Class and family of the cis-regulatory elements identified in putative promoters of the *MKK* genes, indicating their reported biological function, the number of detected motifs, their distribution across *MKK* groups (A-D), and the specific promoters in which they are present.
