# Supplementary figures and images for: Expression pattern of the 10 mitogen-activated protein kinase kinases (MAPKK) encoded on Arabidopsis thaliana genome
Source: Plant Signal Behav. 2026 Jul 6;21(1):2697589. doi: 10.1080/15592324.2026.2697589 (PMC13348940; doi:10.1080/15592324.2026.2697589)

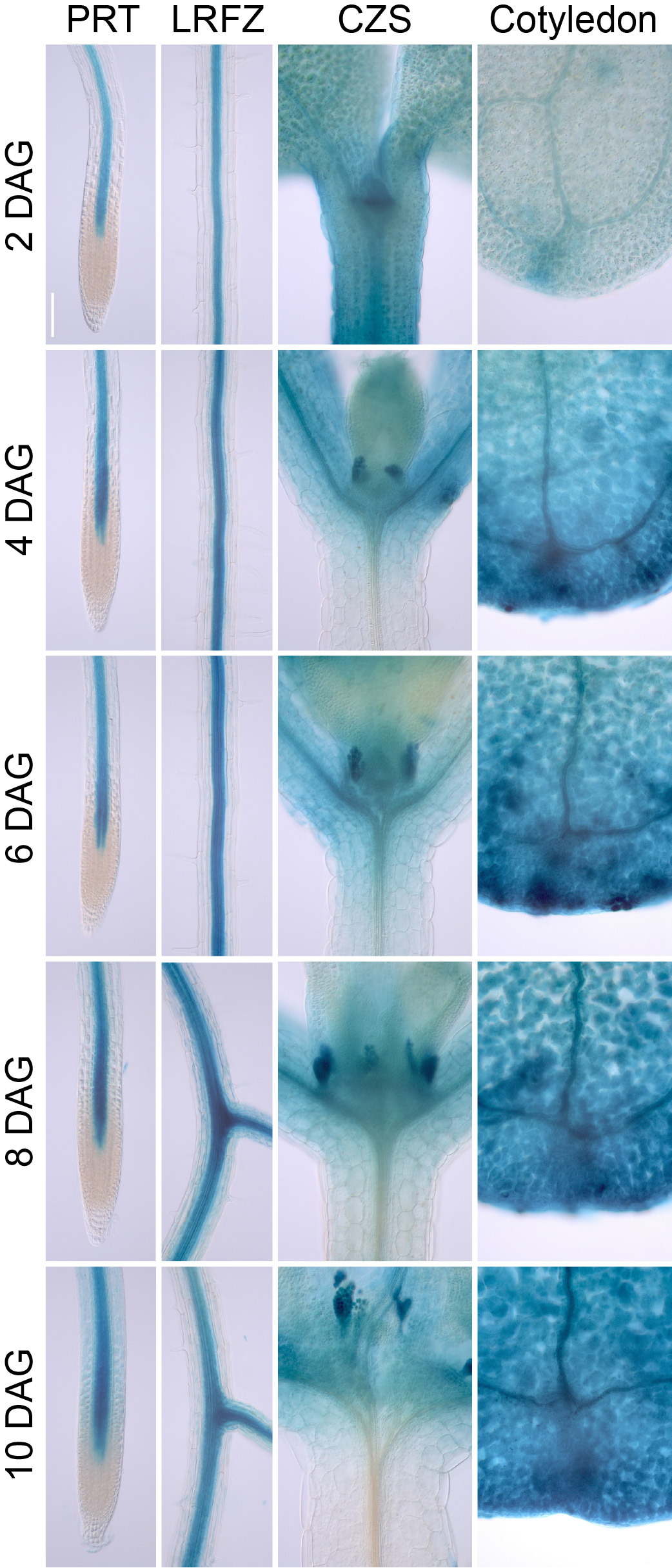

Supplement: Supplementary material — Figure_S5.jpg [file KPSB_A_2697589_SM7549.jpg]

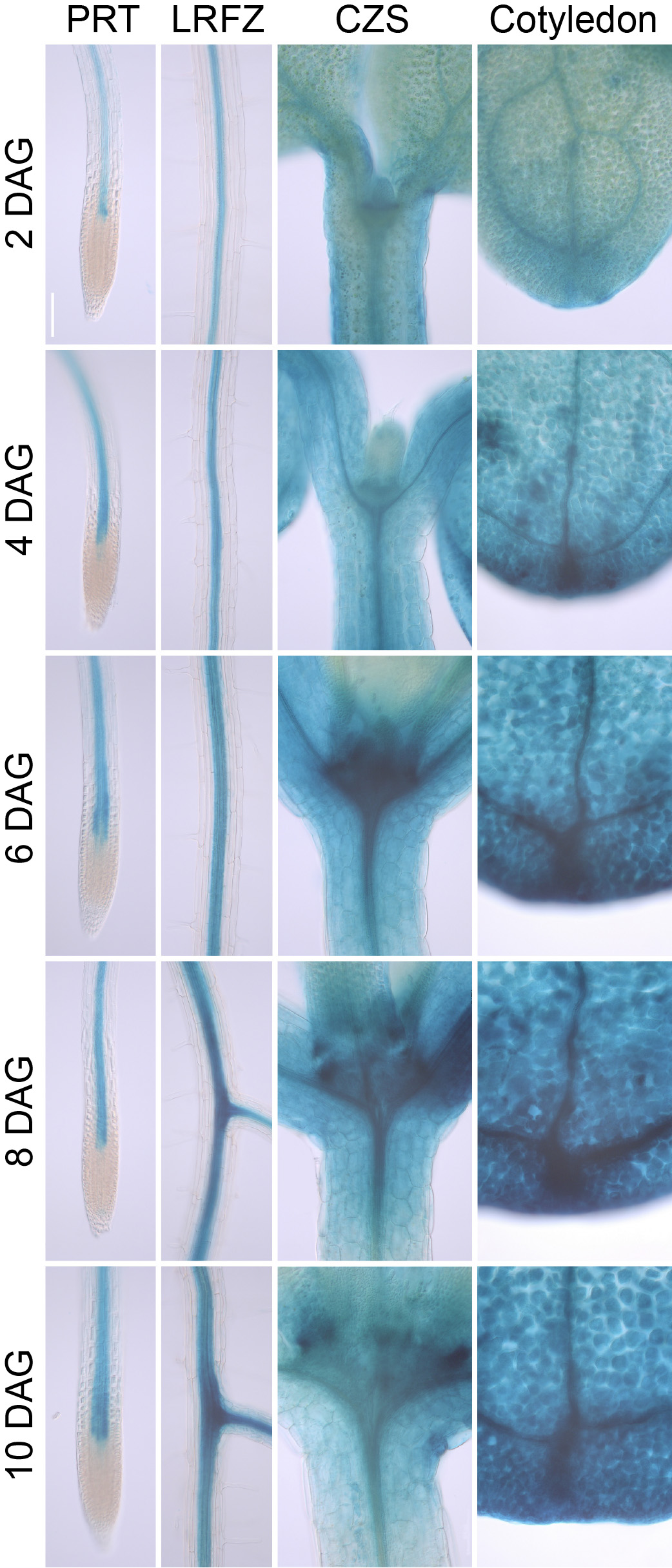

Supplement: Supplementary material — Figure_S2.jpg [file KPSB_A_2697589_SM7550.jpg]

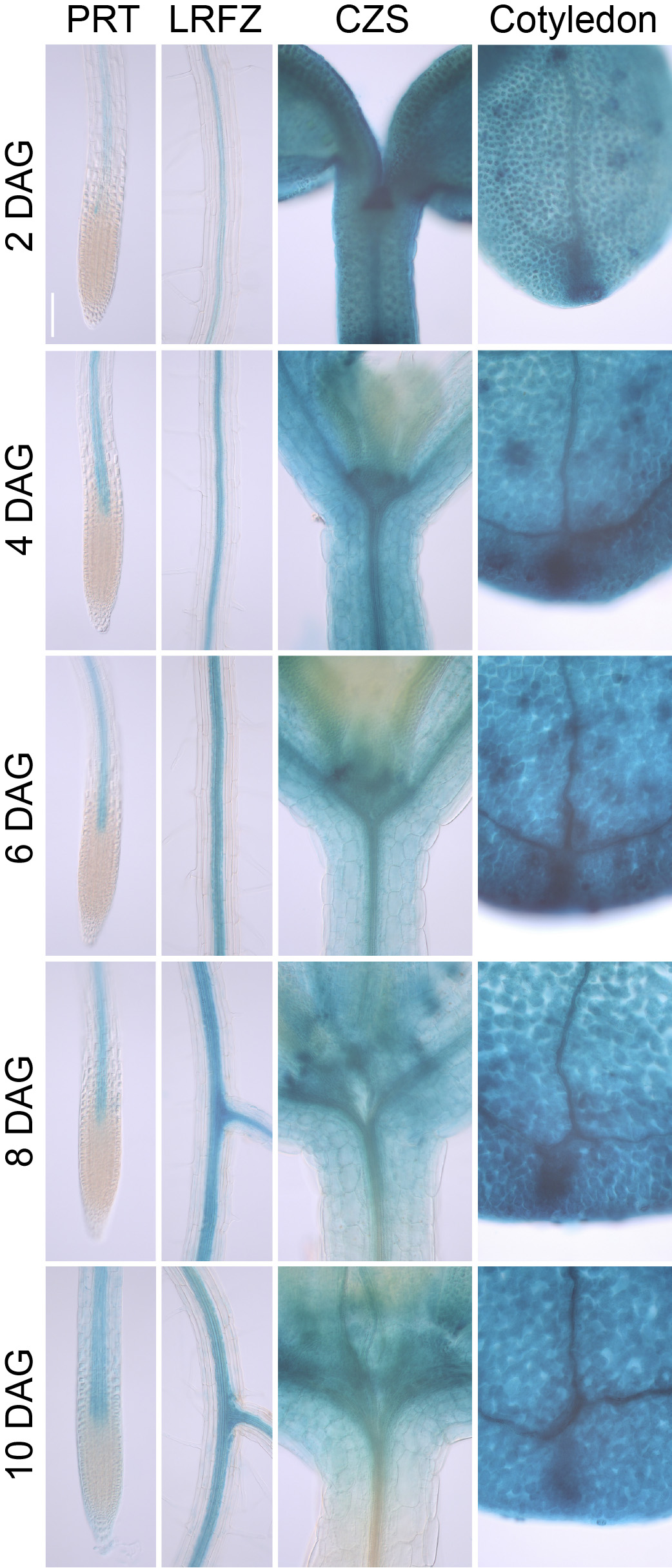

Supplement: Supplementary material — Figure_S4.jpg [file KPSB_A_2697589_SM7552.jpg]

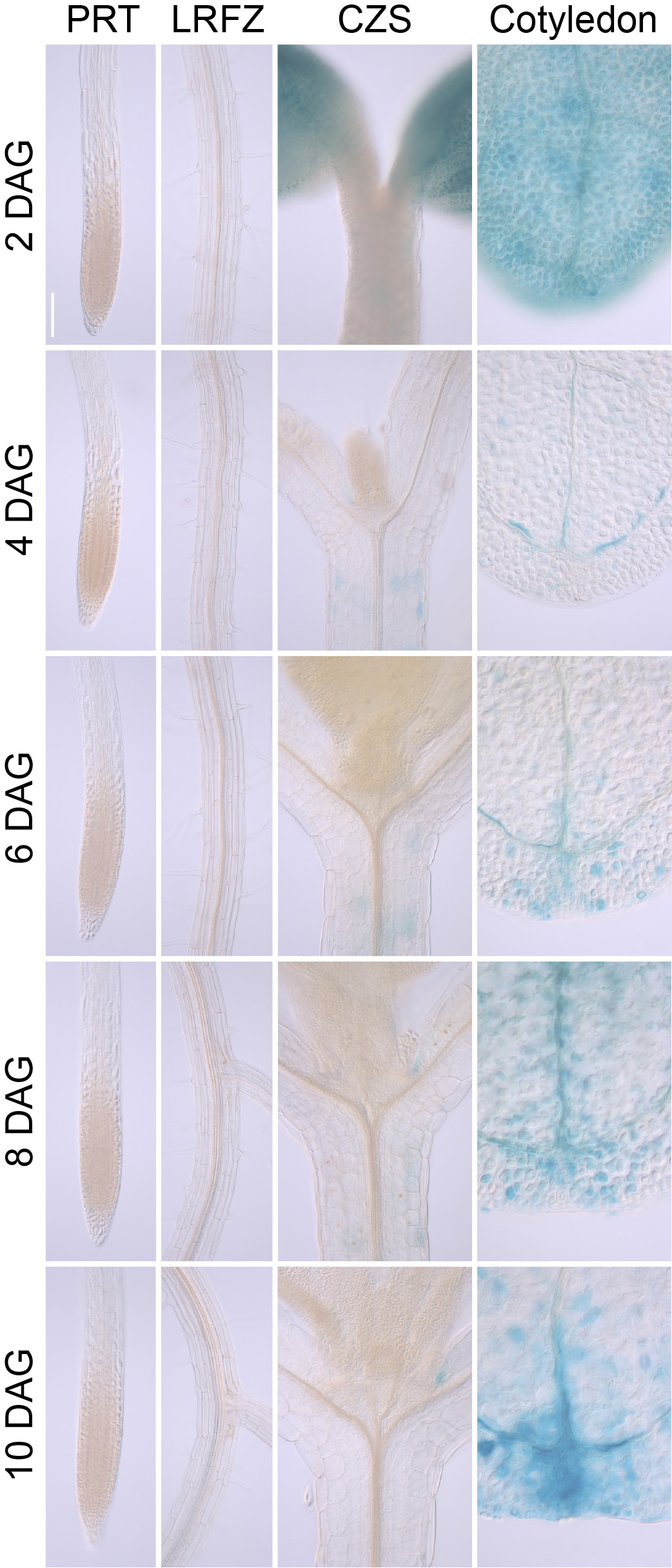

Supplement: Supplementary material — Figure_S6.jpg [file KPSB_A_2697589_SM7553.jpg]

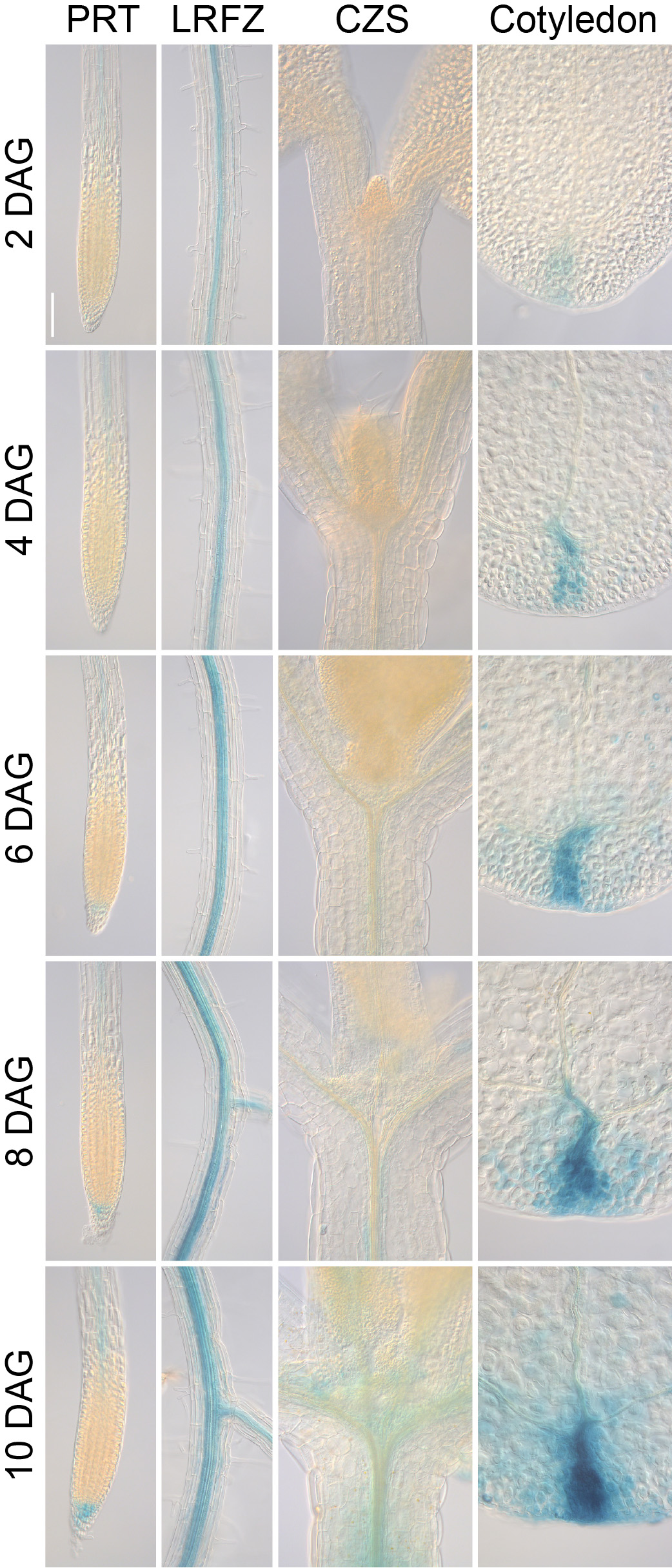

Supplement: Supplementary material — Figure_S3.jpg [file KPSB_A_2697589_SM7554.jpg]

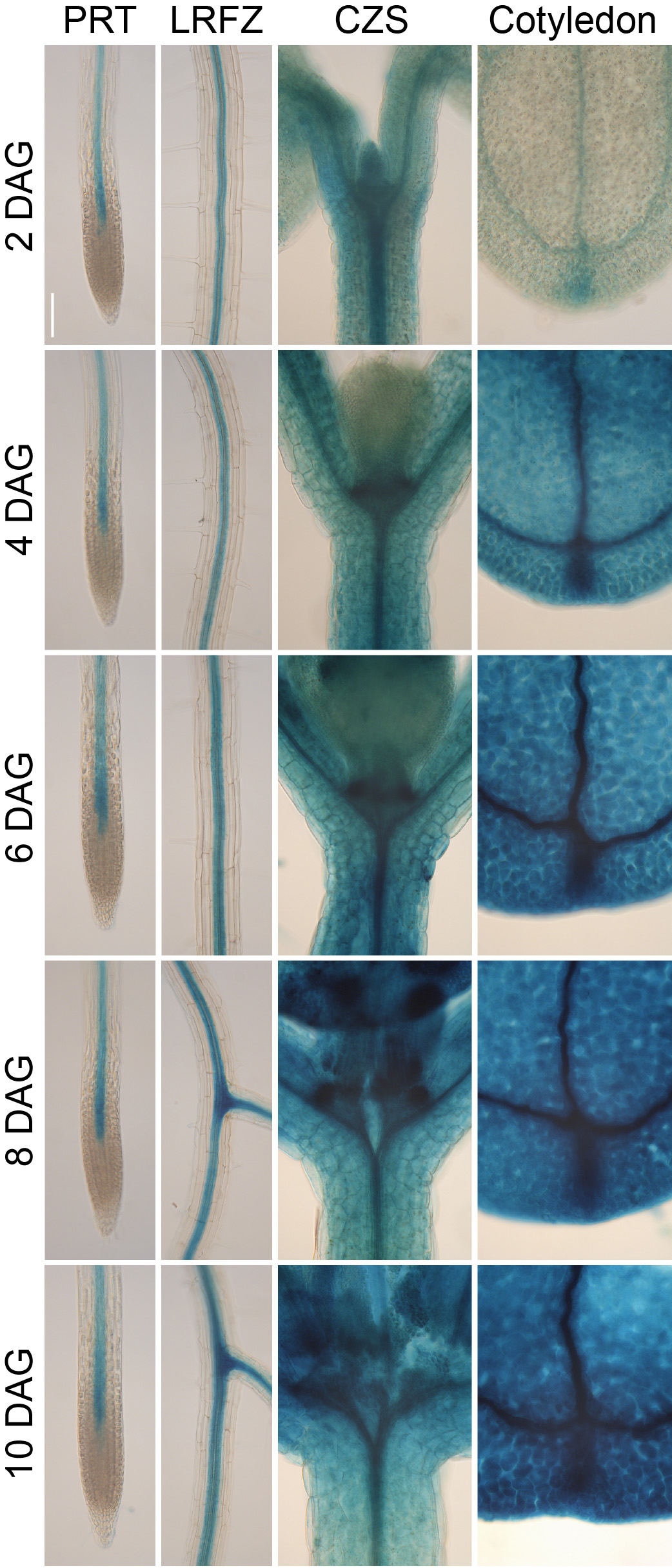

Supplement: Supplementary material — Figure_S1.jpg [file KPSB_A_2697589_SM7555.jpg]

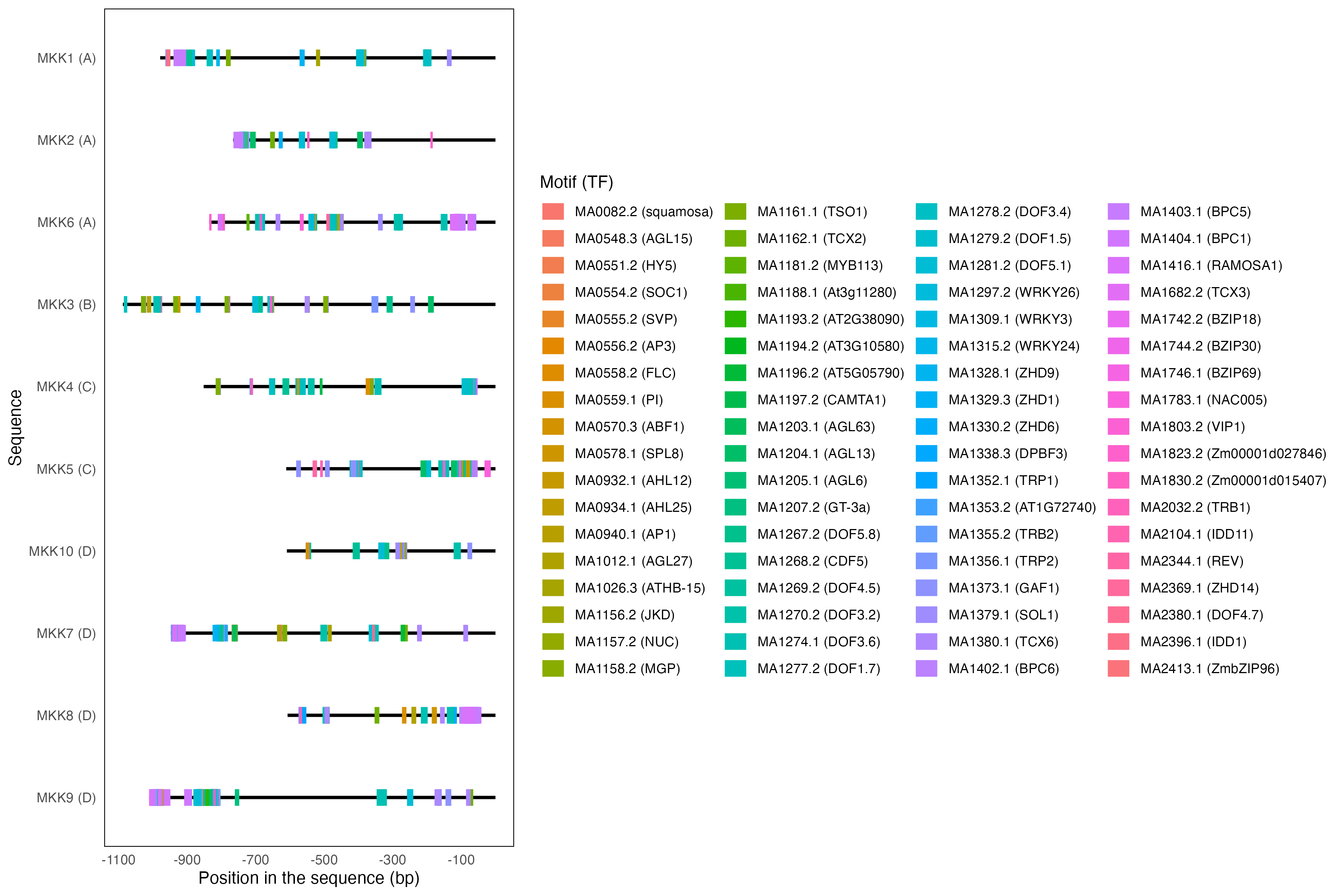

Supplement: Supplementary material — Figure_S7.tiff [file KPSB_A_2697589_SM7551.tif]
